# Genetic and metabolic signatures of *Salmonella enterica* subsp. *enterica* associated with animal sources at the pangenomic scale

**DOI:** 10.1186/s12864-019-6188-x

**Published:** 2019-11-06

**Authors:** Meryl Vila Nova, Kévin Durimel, Kévin La, Arnaud Felten, Philippe Bessières, Michel-Yves Mistou, Mahendra Mariadassou, Nicolas Radomski

**Affiliations:** 1 French Agency for Food, Environmental and Occupational Health and Safety (Anses), Laboratory for Food Safety (LSAL), Paris-Est University, Maisons-Alfort, France; 2 Applied Mathematics and Computer Science, from Genomes to the Environment (MaIAGE), French National Institute for Agricultural Research (INRA), Université Paris-Saclay, 78350 Jouy-en-Josas, France

**Keywords:** Microbial genomics, *Salmonella* adaptation, Genome wide association study, Gene ontology enrichment analysis

## Abstract

**Background:**

*Salmonella enterica* subsp. *enterica* is a public health issue related to food safety, and its adaptation to animal sources remains poorly described at the pangenome scale. Firstly, serovars presenting potential mono- and multi-animal sources were selected from a curated and synthetized subset of Enterobase. The corresponding sequencing reads were downloaded from the European Nucleotide Archive (ENA) providing a balanced dataset of 440 *Salmonella* genomes in terms of serovars and sources (i). Secondly, the coregenome variants and accessory genes were detected (ii). Thirdly, single nucleotide polymorphisms and small insertions/deletions from the coregenome, as well as the accessory genes were associated to animal sources based on a microbial Genome Wide Association Study (GWAS) integrating an advanced correction of the population structure (iii). Lastly, a Gene Ontology Enrichment Analysis (GOEA) was applied to emphasize metabolic pathways mainly impacted by the pangenomic mutations associated to animal sources (iv).

**Results:**

Based on a genome dataset including *Salmonella* serovars from mono- and multi-animal sources (i), 19,130 accessory genes and 178,351 coregenome variants were identified (ii). Among these pangenomic mutations, 52 genomic signatures (iii) and 9 over-enriched metabolic signatures (iv) were associated to avian, bovine, swine and fish sources by GWAS and GOEA, respectively.

**Conclusions:**

Our results suggest that the genetic and metabolic determinants of *Salmonella* adaptation to animal sources may have been driven by the natural feeding environment of the animal, distinct livestock diets modified by human, environmental stimuli, physiological properties of the animal itself, and work habits for health protection of livestock.

## Background

*Salmonella* is one of the main agents of foodborne bacterial infections in human. In particular, *Salmonella enterica subsp. enterica* serovars are responsible for around 80 million foodborne cases annually in developed countries [1, 2]. The 2600 known *S. enterica* subsp. *enterica* serovars exhibit a broad diversity in phenotypes including infectious patterns, lifestyle, reservoirs, vectors and host spectrum [3]. The genomic determinants of these phenotypes remain however partially characterized [4–11]. The present work tackles the genomic and metabolic signatures highlighting the poorly understood mechanisms of adaptation to animal sources at the pangenome scale of *Salmonella enterica subsp. enterica*.

From extremely clonal to the freely recombinant, bacterial evolution is mainly governed by stochastic point mutations induced by replication errors or damage of DNA (i.e. single nucleotide polymorphisms SNPs and small insertions/deletions InDels), and Horizontal Gene Transfers (HGT) promoted by homologous and non-homologous recombination events [12]. The homologous recombination events correspond to the replacement or inversion of identical or similar sequences [13], while the non-homologous recombination refers to the incorporation of new genetic material between distinct genomes [12]. The HGT whose large fragments are also named Mobile Genetic Elements (MGEs), can occur in bacterial genomes during transformation (i.e. transfer of pathogenicity islands, transposons or insertion sequences between two bacterial chromosomes), conjugation (i.e. transfer of plasmids between two bacterial genomes) and transduction (i.e. transfer and/or chromosomal incorporation of phages into bacterial genomes) [12].

The molecular mechanisms of host adaptation driven by the evolution were revealed by conventional molecular biology highlighting that *S. enterica* subsp. *enterica* extended over a wide range of hosts including birds, fishes, reptiles, amphibians, bovines, pigs and others [14]. Since the divergence from the most recent common ancestor (MRCA) with *Escherichia coli* approximately 100–160 million years ago [15], the coevolution of *Salmonella* and animal hosts during millions of years, has led to the acquisition of genes required for intestinal infection (i.e. *S. bongori* species), colonization of deeper tissues (i.e. other *S. enterica* subspp.), and expansion toward warm-blooded vertebrates (i.e. *S. enterica* subsp. *enterica*) [16]. The adaptation to warm-blooded animals started by generalist host associations related to gastrointestinal infections and transmission induced by the short-term proliferation in the intestine, or independently of the replication in the intestine by dissemination and persistence in systemic niches that are devoid of competing microbiota and can last for the lifetime of the hosts [17].

Without exhaustive data for all known serovars of *S. enterica* subsp. *enterica*, some are considered to be more adapted to mono-hosts, like Gallinarum in avian [4, 7, 10] or Dublin in bovine [4, 6]. The evolution of *S. enterica* subsp. *enterica* within hosts may have led some serovars to specialize to their host. This adaptation is accompanied by loss of bacterial fitness for inter-host transmission and apparent convergence in pathogenesis [17]. For instance, Typhi and Paratyphi A cause typhoid and paratyphoid in human, Gallinarum is associated with fowl typhoid, Abortusovis induces abortion in sheep, and Dublin and Choleraesuis are involved in bacteraemia of cattle and pigs, respectively [17]. Even if most of studies focusing on transformed seafood products [18, 19] do not provide prevalence of infected fish *in natura* [20], the serovar Bareilly is also supposed to be adapted to fish. Causing gastroenteritis, other serovars are also considered as adapted to multiple hosts like Typhimurium [9, 21] or Enteritidis [11].

Most of studies based on conventional molecular biology demonstrated that acquisition by HGT of *Salmonella* Pathogenicity Islands (SPIs) that contain genes coding for invasion, survival, and extraintestinal spread is among the prominent molecular mechanisms explaining the host adaptation of *S. enterica* subsp. *enterica* [22]. The 23 known SPIs are mainly involved in adhesion to epithelial cells (i.e. SPI-3, 4 and 5), invasion in their *Salmonella* containing vacuoles (SCV) (i.e. SPI-1 and 14), resistance to overcoming colonization of the intestinal mucus layer (i.e. SPI-6), induction of inflammation and neutrophil recruitment (i.e. SPI-1), as well as survival (SPI-11, 12 and 16) and outer membrane remodeling (SPI-2, 5 and 13) when they are in macrophages [23–25]. More precisely, two type III secretion systems (i.e. T3SS-1 and T3SS-2) encoded on SPI-1 and SPI-2 allow invasion of host epithelium and intracellular survival, respectively [17]. It must also be noted that the prophages Gifsy-2 and Fels-1 are involved in resistance to oxidative stress from neutrophils during infection, while the prophages Gifsy-1 and sopEФ induce downregulation of inflammation in SCV and robust inflammation of the epithelial cells, respectively [25].

Albeit host adaptation of *S. enterica* subsp. *enterica* is poorly described at the genomic scale [4–11], the studies focusing on its accessory genome, confirmed that SPIs play a major role in the adaptation of few serovars to avian (e.g. SPI19 in Gallinarum and Pullorum [7, 10]) and bovine (e.g. SPI6 and SPI7 in Dublin [4, 7]) hosts. These studies emphasized that plasmids are also a major determinant explaining adaptation to avian (e.g. resistance-virulence plasmid of Kentucky [5]) and bovine (e.g. plasmid pSDV of Dublin [6]). The unique study focusing on the coregenome demonstrated that the divergence, probably induced by animal diet, between mammalian-host adapted Dublin and multi-host adapted Enteritidis was due to fixed variants targeting regions involved in metabolic pathways of amino acids linked to glutamate [11]. This study also showed that limited ion supply in avian tract and L-arginine used for growth of laying hens, implied modifications of ion transport (i.e. potassium-efflux system in Gallinarum) and L-arginine catabolism (i.e. alanine racemase in Pullorum) of avian-adapted serovars [11].

The Genome Wide Association Study (GWAS) aims to identify the genetic variations associated with particular phenotypic traits within a population [26]. Following the first tool computing GWAS with a correction of Eukaryotic population structure based on SNPs (PLINK) [27], combinations of different methods have been implemented in the recently developed microbial GWAS. Over the last 10 years, microbial GWAS was implemented to explore a diversity of biological problems: genetic backgrounds of microbial origin [28], persistence [29], host preference [30], virulence [31, 32], and antibiotic resistance [33–42]. In comparison to human GWAS, the confounding factors of the microbial GWAS include genome selection, homologous recombination events, population structure, as well as genome wide significance [43]. Microbial GWAS takes into account these confounding factors and tests for associations between mutations and phenotypes of interest [40, 43–50]. In a context of source tracking for food safety [1, 2], microbial GWAS seems a promising tool to identify mutations associated to animal sources in order to improve models of source attribution [51].

Compared to the 10 years of developments focusing on microbial GWAS, Gene Ontology Enrichment Analysis (GOEA) has been undergoing constant improvements since the beginning of the twenty-first century and recently reached maturity for bacteria. GOEA is indeed rarely applied to bacterial genomes in spite of successful studies applying this approach to decipher host adaptation of *S. enterica* at the coregenome level [11], compare transcriptome expression profiles of minimally and highly pathogenic *S. enterica* [52], or cluster orthologous groups among differentially expressed microbial genes [53]. The GOEA proposes to test the hypergeometric distributions of GO-terms from a list of interest (i.e. tested sample) with regards to a broader set of GO-terms (i.e. universe) based on the assumption of dependencies between the GO-terms implemented through a parent-child approach [54]. GOEA was historically proposed by the Gene Ontology Consortium [55] and is today centralized in the universal protein knowledgebase commonly known as UniProt [56]. More precisely, the GO-terms link the genes and/or variants to the metabolic pathways [57] and are synthetized through a directed acyclic graph (DAG) of GO-terms into three independent ontologies called biological process (BP), molecular function (MF) and cellular component (CC) [55].

Taking into account confounding factors (i.e. genome selection, homologous recombination events, population structure and genome wide significance), the present study proposes to decipher *Salmonella* adaptation to animal sources (i.e. avian, bovine, swine and fish) based on microbial GWAS implementing accessory genes and coregenome variants (i.e. SNPs and InDels), as well as an advanced population structure correction [40]. The mutations (i.e. genes and variants) associated to traits of interest (i.e. avian, bovine, swine and fish sources) were also linked to metabolic pathways by GOEA implementing a parent-child approach [11]. To our knowledge, the present study is the first to apply successively microbial GWAS and GOEA at the pangenome scale.

## Results

### Distributions of serovars from potential mono-and multi-animal sources

**Fig. 1:**
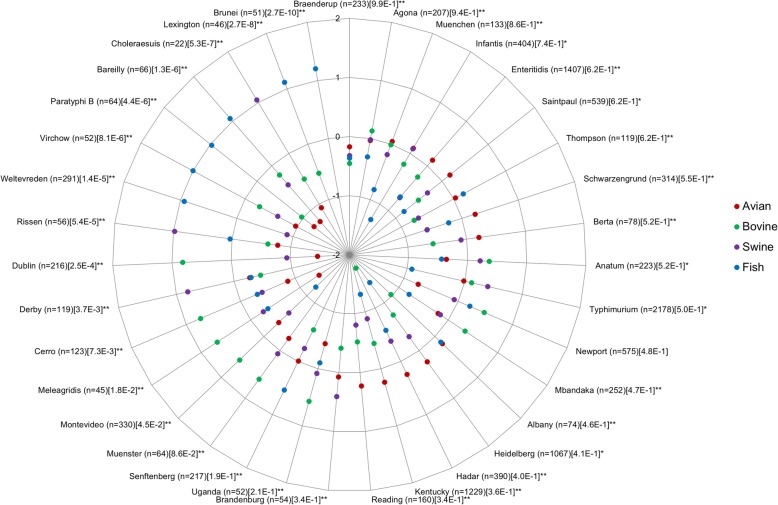
Relative proportions of serovars of *Salmonella enterica* subsp. *enterica* found in each animal source (i.e. avian, bovine, fish and swine) in log-scale and corrected by the baseline proportions in the curated subset of Enterobase (see text for details). The present study focusing on adaptation to animal sources (*n* = 13,635) does not include isolates from environment, composite foods of the retail market and humans, which are considered as vectors of pathogen expositions and exposed susceptible consumers, respectively. The indexes higher and lower than zero represent sources in which serovars are over- and under-represented, respectively. The total effectives and *p*-values of Chi-square tests of uniformity applied to indexes are in brackets and square brackets, respectively. The serovars are sorted from the lowest (i.e. potentially mono-animal source) to highest (i.e. potentially multi-animal source) *p*-values. An asterisk stands for less than 20 samples from fish. A double asterisk stands for less than 20 samples from avian, bovine, swine and fish sources

The composition of *Salmonella* serovars from EnteroBase [58] were investigated in order to build a genome dataset taking into account the confounding factors of microbial GWAS (Additional file 1), namely genome selection [43, 44], recombination [43, 45–47], population structure [33, 40, 43, 48] and genome wide significance [43, 50]. Out of 13,635 records from a curated and synthetic subset of Enterobase, *Salmonella* isolates were mainly distributed in avian, bovine, fish, plant, shellfish and swine sources, enabling the selection of multiple strains for each studied serovar and source when building our dataset (Additional file 2). Because the detailed records from Enterobase were not enough detailed to determine if the strains from plants and shellfishes were isolated inside or outside tissues, the present study focuses on adaption to the following sources: avian, bovine, swine and fish. Among strains isolated from these sources (*n* = 11,450), most (22 out of 35) serovars (Fig. 1) had single animal sources (*p* < 4.5 × 10^− 1^, Chi-square tests of uniformity to find serovars associated with some sources). Respecting high levels of diversity in terms of phylogenomic relationships in agreement with previous studies [59], geographical origins, dates of isolation and BioProject accession numbers, a balanced dataset of serovars from putative mono- and multi-animal sources (Fig. 1) were selected. This dataset was used to detect mutations and metabolic pathways associated with the adaptation of *Salmonella* serovars to their animal sources. More precisely, isolates of the *Salmonella* serovars Newport, Typhimurium and Anatum were selected as multi-animal sources, whereas other serovars were selected as mono-animal sources related to avian (i.e. Heidelberg, Kentucky, Hadar), bovine (i.e. Dublin, Cerro, Meleagridis), swine (i.e. Chloraesuis, Rissen, Derby) or fish (i.e. Brunei, Lexington, Bareilly) (Additional file 3).

### Authenticity and completeness of detected mutations

Among the 440 selected isolates, we replaced 25 strains for which paired-end reads presenting signs of exogenous DNA and inconsistencies between in vitro (i.e. sero-agglutination register in Enterobase) [60] and in silico (i.e. SISTR program) identifications of serovars [61]. The absence of exogenous DNA was checked based on the distribution of GC% (i.e. 52.12 ± 0.09) and total sizes of studied draft genomes (i.e. Additional file 4) in comparison with the complete circular genomes selected as references during the scaffolding steps (i.e. 4.73 ± 0.16 × 10^− 6^; *n* = 74).

**Fig. 2:**
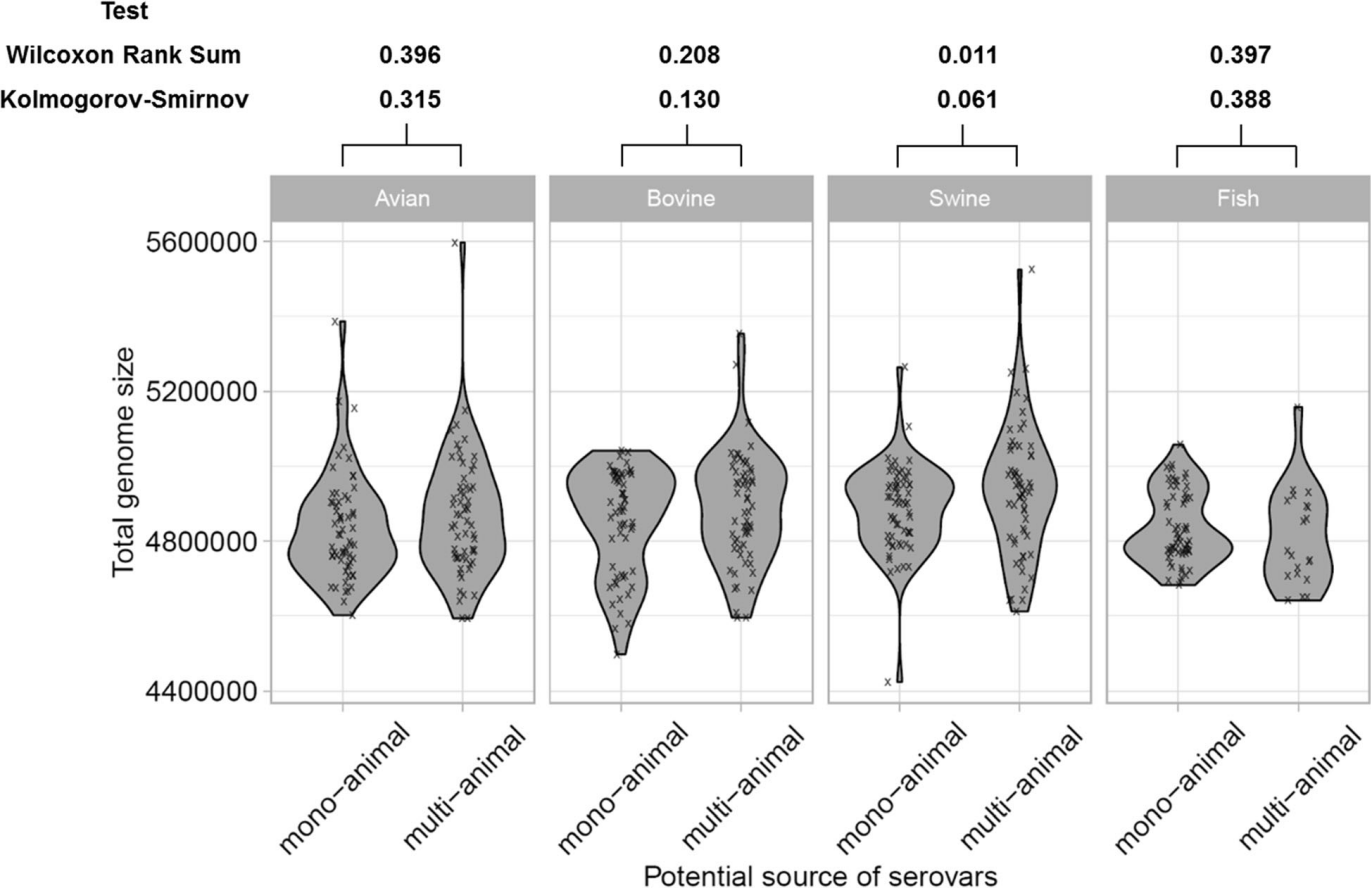
Total genome sizes of *Salmonella enterica* subsp. *enterica* serovars isolated from potential mono- and multi-animal sources related to avian (*n* = 120), bovine (*n* = 120), swine (*n* = 120) and fish (*n* = 80). Based on a curated and synthetic dataset of Enterobase, the *Salmonella* serovars Newport, Typhimurium and Anatum were selected and considered as serovars from potential multi-animal sources. The other selected serovars were considered as serovars from potential mono-animal sources related to avian (i.e. Heidelberg, Kentucky, Hadar), bovine (i.e. Dublin, Cerro, Meleagridis), swine (i.e. Chloraesuis, Rissen, Derby) and fish (i.e. Brunei, Lexington, Bareilly). Normality of the data was checked using Shapiro-Wilk test (*p* < 1.0 × 10^− 2^). The statistical differences in terms of median and distribution were assessed by non-parametric Wilcoxon rank sum and Kolmogorov-Smirnov tests, respectively

The sizes of these 440 draft genomes (Fig. 2) agreed with the literature and ranged from 3.39 to 5.59 Mbp (i.e. between 3969 and 9898 genes) [62]. In line with studies emphasizing that host adaptation and increased pathogenicity of *Salmonella* serovars are not necessarily reflected in smaller genome sizes [5], we did not detect significant differences in terms of median values and distributions of total genomes sizes (Fig. 2) between strains from mono- and multi-animal sources (Fig. 1).

NG50 values close to the sizes of the reference circular genomes, low number of long scaffolds (i.e. between 1 and 83 higher than 1000 bp), and almost complete genome fractions (i.e. ≈ 100%) (Additional file 4), were considered as evidences of assembly quality sufficiently high to perform pangenome extraction [63]. The pangenome extraction revealed logarithmic and hyperbolic forms of curves representing the new and conserved genes according to the sizes of genome dataset, respectively (Additional file 4). According to previous studies that estimated strict coregenome sizes of *Salmonella* between 1500 [64] and 2800 [65] genes, the present open pangenome of *Salmonella enterica* consists in 2705 core genes and 19,130 accessory genes. Given the high breadth (i.e. ≈ 100%) and depth coverages (i.e. > 30X) (Additional file 4), we performed variant calling analysis based on reference mapping [66]. Overall, 178,351 variants (98% of SNPs and 2% of InDels) were detected in the coregenome, including 139,514 variants from 3030 homologous recombination events. These accessory genes and coregenome variants were considered as genuine mutations, as the analysis followed best practices for genome assembly [63] and variant calling [66].

### Congruencies of phylogenomic reconstructions

Visual inspections of the few incongruencies between the phylogenomic trees obtained from 3 different approaches, namely ‘variants including homologous recombination events’ (called A), ‘variants excluding homologous recombination events’ (called B) and ‘concatenated orthologous genes’ (called C) (Additional file 5), are in accordance with the high congruencies of pairwise distances emphasized by the corresponding cophenetic correlation coefficients (Table 1). Even though the trees have some branches in conflicts (see Robinson-Foulds indexes in Table 1), the few incongruencies result from a Subtree Prune Regrafting move and the topologies are globally congruent (see Fowlkes-Mallows indexes in Table 1). Swapped nodes are present comparing the serovars Typhimurim and Heidelberg to Anatum (A versus C), Bareilly (B versus C), or Anatum and Bareilly (A versus B) (Additional file 5). Considering the high level of agreement between the phylogenies, (Table 1 and Additional file 5) and following the recommendations of Hedge and Wilson [67], the present study will discuss the adaptation to animal sources mainly based on the tree retaining most of genetic information (i.e. reconstructed from the approach ‘A’). The phylogenomic reconstruction from the approach ‘A’ (i.e. iVarCall2) was indeed inferred based on coregenome SNPs from intra- and intergenic regions, as well as homologous recombination events, contrary to the approaches ‘B’ (i.e. ‘variants excluding homologous recombination events’ from iVarCall2 and ClonalFrameML) and ‘C’ (i.e. ‘concatenated orthologous genes’ from Roary).

**Table 1:** Congruency parameters between phylogenomic reconstructions of strains belonging to different serovars of *Salmonella enterica* subsp. *enterica* (*n* = 440) in terms of distance and topology. The phylogenomic reconstructions were performed by maximum likelihood selecting the most appropriate models of evolution and checking ultrafast bootstrap convergences (i.e. IQ-Tree). The compared approaches ‘variants’ and ‘genes’ correspond to phylogenomic trees reconstructed using pseudogenomes from variant calling analysis (i.e. iVARCall2) including (A) or excluding (B) variants from recombination events (i.e. ClonalFrameML), and concatenated orthologous genes (C) from pangenome analysis (i.e. Roary), respectively. The cophenetic function of the ‘dendextend’ R package was used to compute the cophenetic correlations. The dendrogram function of the ‘dendextend’ R package was used to compute the Fowlkes-Mallows indexes. The treedist function of the ‘phangorn’ R package was used to compute the Robinson-Foulds indexes

Tree parameters ^a^	Congruency parameters	Compared approaches of phylogenomic reconstructions		
		‘A’ vs ‘B’	‘C’ vs ‘A’	‘C’ vs ‘B’
Distance	Cophenetic correlation (Pearson)	0.989	0.993	0.981
	Cophenetic correlation (Kendall)	0.766	0.828	0.742
	Cophenetic correlation (Spearman)	0.924	0.954	0.911
Topology	Fowlkes-Mallows index	0.650	0.600	0.600
	Robinson-Foulds index	370	264	410

^a^ distance refers to similarity between trees in terms of correlation between the cophenetic distance matrices. Topology refers to differences between two trees in terms of node clustering, respectively

### Phylogenomic relationships between serovars from potential mono- and multi-animal sources

**Fig. 3:**
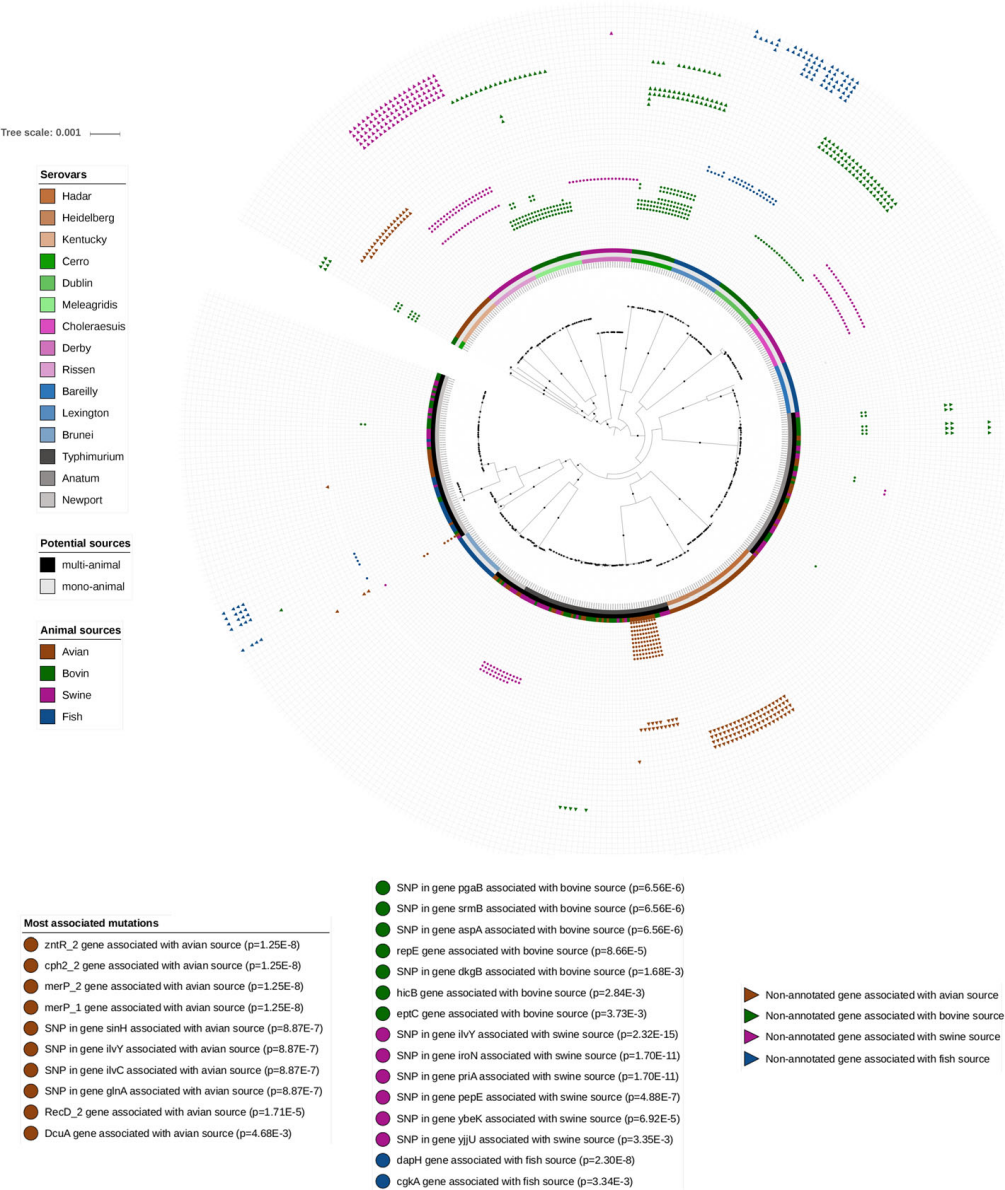
Maximum likelihood phylogenomic tree of *Salmonella enterica* subsp. *enterica* serovars (*n* = 440) from potential mono- and multi-animal sources. Based on pseudogenomes inferred with the variant calling workflow iVARCall2, the workflow IQ-Tree selected the most appropriate model of evolution (GTR + I + G4) according to Akaike Information Criteria (AIC) and reconstructed the tree with an ultrafast approximation of phylogenomic bootstrap. The present phylogenomic tree was inferred including SNPs from recombination events and was rooted using the most closely related *indica* subspecies as an outgroup. The potential mono- and multi-animal sources were assigned based on Chi-square tests of uniformity applied on a curated and synthetic subset of Enterobase. Examples of mutations associated with animal sources by microbial GWAS are presented (i.e. Wald tests). These associated mutations refer to polygenicity with regard to Quantile-Quantile (QQ) plots from microbial GWAS (i.e. *p* < 1 × 10^− 2^) and present high (i.e. > 5%) and low (i.e. < 5‰) frequencies of presence (i.e. genes and alternative variants) in the studied and compared genomes, respectively. The serovars (i.e. colored squares), potential sources (i.e. black and grew squares), animal sources (i.e. colored squares), as well as annotated (i.e. colored circles) and non-annotated (i.e. colored triangles) mutations associated to animal sources, are represented from the internal to external rings. The colored circles and triangles represent present genes or alternative variants, whereas missing data refers to absente genes or reference variants, respectively. Most of the branches of the tree (i.e. 85%) are supported by bootstrap values higher than 90% (i.e. black circles) and the corresponding newick file is accessible under request

With the exception of serovars Newport and Cerro, all other serovars were monophyletic (Fig. 3) in all trees (Additional file 5). While the genomes of serovars from multi-animal sources were clustered into three distinct phylogenomic clusters (i.e. first lineage of Newport versus second lineage of Newport and Typhimurium versus Anatum), those from mono-animal sources were grouped by serovar (Fig. 3). The coexistence of purely clonal (i.e. mono-animal sources) and nearly panmictic (i.e. multi-animal sources) serovars (Fig. 3), emphasizes the necessity to correct the population structure when performing a microbial GWAS (Additional file 1) to find mutations associated to animal sources (i.e. avian, bovine, swine and fish).

### Consideration of confounding factors during microbial GWAS

With the objective to take into account the confounding factors during microbial GWAS (Additional file 1), we compared different dataset of genomes to assess the correction of population structure and estimated the impact of the homologous recombination events [43]. More precisely, 9 microbial GWAS were performed for each animal sources (i.e. 36 analyses) considering different datasets of genomes from multi- (i.e. panmictic expansion) and/or mono- (i.e. clonal expansion) animal sources in the cluster presenting the phenotype of interest, as well as the cluster without this latter one (Additional file 6). Excluding the variants from homologous recombination events, 9 other microbial GWAS (i.e. 36 analyses) were performed with these different datasets of genomes (Additional file 7). Probably due to the coexistence of purely clonal to nearly panmictic lineages in the dataset of 440 genomes (Additional file 1), the datasets of genomes and variants from homologous recombination events affected the population structure corrections (Additional files 6 and 7). Expected shapes of quantile-quantile (QQ) plots referring to suitable population structure corrections (i.e. inflation for only highly significant observed *p*-values) were systematically checked including genomes from mono- and multi-animal sources in both studied strains and compared strains for the avian, bovine, swine and fish sources (Additional files 6 and 7). Concerning these expected shapes of QQ plots presenting inflations for only highly significant observed *p*-values, much more stratification of causal mutations were observed including variants from homologous recombination events (Additional file 6), compared to microbial GWAS excluding them (Additional file 7). All the 440 genomes included, we observed that most of the associated mutations were different comparing microbial GWAS performed with and without variants from recombination events (Table 2). According to this observation and the authors suspecting the homologous recombination events to conceal the detection of causal variants by microbial GWAS [43, 45–47], we decided to exclude the coregenome variants from these regions during microbial GWAS (i.e. 139,514 variants from 3030 homologous recombination events). Taking into account all the known confounding factors (Additional file 1), and even if common genome wide significance of human GWAS is around *p* ≤ 1 × 10^− 6^, the polygenicity was estimated at *p* ≤ 1 × 10^− 2^ according to the QQ plots of the present study focusing on microbial GWAS (Additional file 7). Without consensus concerning the genome wide significance of microbial GWAS [43], and with regards to frequencies of presence and absence of genes and alternative variants (Additional file 8), we estimated and checked visually that associated mutations present *p*-values of association between *p* = 8.78 × 10^− 3^ and *p* = 2.32 × 10^− 15^ (Fig. 3 and Additional file 8). These mutations associated by microbial GWAS have been retained to apply downstream GOEA.

**Table 2:** Mutations of *Salmonella enterica* subsp. *enterica* serovars (*n* = 440) associated with animal sources (i.e. avian bovine, swine and fish) by microbial GWAS including or excluding variants from recombination events. The accessory genes and coregenome variants (i.e. SNPs and InDels) were annotated with Prokka (1.12) and SNPeff (4.1 g), respectively. After potential exclusion of variants from recombination events based on iVARCall2 and ClonalFrameML, the workflow ‘microbial-GWAS’ corrects the population structure based on Linear Mixed Model (LMM), then perform associations with Wald tests implemented in GEMMA. The associated mutations (i.e. Wald tests) refer to polygenicity with regard to Quantile-Quantile (QQ) plots from microbial GWAS (i.e. *p* < 1 × 10^− 3^ and *p* < 1 × 10^− 2^, with or without recombination events) and present high (i.e. > 5%) and low (i.e. < 5‰) frequencies of presence (i.e. genes and alternative variants) in the studied and compared genomes, respectively

Animal source	Comparison of associated mutations from microbial GWAS			
	Including homologous recombination		Excluding homologous recombination	
	All	Unique	All	Unique
avian	41	36	18	13
bovine	21	18	16	13
swine	35	30	11	6
fish	5	4	7	6

### Mutation associated with animal sources (i.e. microbial GWAS)

No matter the phenotype of interest, only partial associated mutations were detected by microbial GWAS (Fig. 3). While the presence of genes and presence of alternative variants were associated with animal sources, the absence of genes and presence of reference variants were not associated with animal sources. This observation is in accordance with the fact that losses of unessential functions do not necessarily refer to the adaptation to animal sources, as previously reported [12], or unconfirmed [5], concerning the host adaptation and restricted host transmission. As suspected with regard to higher functional impacts of accessory genes compared to coregenome variants, 38 genes were detected as associated with animal sources, whereas only 3 intergenic, 3 synonymous and 8 non-synonymous variants (SNPs and InDels) were associated to these traits of interest (Table 3). Due to the fact that synonymous variants associated to traits of interest (Table 3) may emphasize elements of regulation [68] or phenotypical impacts [69], we decided to retain them in GOEA. To summarize, 38, 34, 26 and 14 associated mutations were detected as signatures of avian, bovine, swine and fish sources, respectively (Additional file 8). Among the latter, annotations are available for only 10, 7, 6 and 2 mutations associated with avian, bovine, swine and fish sources, respectively (Tables 3 and 4).

**Table 3:** Mutations before and after microbial GWAS aiming to associate animal sources (i.e. avian bovine, swine and fish) with mutations from accessory (i.e. genes) and coregenome (i.e. SNPs and InDels) of *Salmonella enterica* subsp. *enterica* serovars (*n* = 440). The accessory genes and coregenome variants (i.e. SNPs and InDels) were annotated with Prokka (1.12) and SNPeff (4.1 g), respectively. After exclusion of variants from recombination events based on iVARCall2 and ClonalFrameML, the workflow ‘microbial-GWAS’ corrects the population structure based on Linear Mixed Model (LMM), then perform associations with Wald tests implemented in GEMMA. The associated mutations (i.e. Wald tests) refer to polygenicity with regard to Quantile-Quantile (QQ) plots from microbial GWAS (i.e. *p* < 1 × 10^− 2^) and present high (i.e. > 5%) and low (i.e. < 5‰) frequencies of presence (i.e. genes and alternative variants) in the studied and compared genomes, respectively

Mutations	Annotations			Before GWAS		After GWAS			
				Including homologous recombination	Excluding homologous recombination	Avian source	Bovine source	Swine source	Fish source
accessory genes and variants	annotated and hypothetical			178,351	38,837	38	34	26	14
accessory genes	annotated			6387	6387	6	3	0	2
	hypothetical			12,743	12,743	8	9	5	5
coregenome variants	intergenic			17,362	2288	1	1	1	0
	intragenic	synonymous		68,157	8365	1	1	1	0
		non synonymous	missenses	65,044	8017	2	2	4	0
			start lost	144	19	0	0	0	0
			stop gained	4202	525	0	0	0	0
			frameshift	1019	136	0	0	0	0
			disruptive inframe insertions	122	14	0	0	0	0
			disruptive inframe deletions	204	31	0	0	0	0
	multiple annotations			2967	312	0	0	0	0

**Table 4:** Functionally annotated mutations (i.e. excluding genes coding hypothetical proteins) of *Salmonella enterica* subsp. *enterica* serovars (i.e. SNPs, InDels and genes) associated by microbial GWAS with animal sources (i.e. avian bovine, swine and fish). The accessory genes and coregenome variants (i.e. SNPs and InDels) were annotated with Prokka (1.12) and SNPeff (4.1 g), respectively. After exclusion of variants from recombination events based on iVARCall2 and ClonalFrameML, the workflow ‘microbial-GWAS’ corrects the population structure based on Linear Mixed Model (LMM), then perform associations with Wald tests implemented in GEMMA. The associated mutations (i.e. Wald tests) refer to polygenicity with regard to Quantile-Quantile (QQ) plots from microbial GWAS (i.e. *p* < 1 × 10^− 2^) and present high (i.e. > 5%) and low (i.e. < 5‰) frequencies of presence (i.e. genes and alternative variants) in the studied and compared genomes, respectively. The genes with undefined names are assigned to STM identifiers with regard to the reference genome of *Salmonella* Typhimurium LT2 (NCBI NC_003197.1). HGVS stands for Human Genome Variation Society. N/A and ND stand for not applicable and not determined. N/A refers to intergenic regions. The term ‘gene’ refers to the gene presence

Studied animal source	Mutation	*p*-value (Wald test)	Gene name	Annotation	Variant position	HGVS notation (DNA)	HGVS notation (protein)	UniprotKB
Avian	Gene	1.2 × 10^− 8^	*zntR2*	HTH-type transcriptional regulator ZntR	N/A	N/A	N/A	P0ACS5
Avian	Gene	1.2 × 10^−8^	*cph2_2*	Phytochrome-like protein cph2	N/A	N/A	N/A	Q55434
Avian	Gene	1.2 × 10^−8^	*merP2*	Mercuric transport protein periplasmic component	N/A	N/A	N/A	P13113
Avian	Gene	1.2 × 10^−8^	*merP1*	Mercuric transport protein periplasmic component	N/A	N/A	N/A	P13113
Avian	Gene	1.7 × 10^−5^	*recD2*	ATP-dependent RecD-like DNA helicase	N/A	N/A	N/A	Q9RT63
Avian	Gene	4.6 × 10^−3^	*dcuA*	Anaerobic C4-dicarboxylate transporter DcuA	N/A	N/A	N/A	P0ABN5
Avian	SNP	8.8 × 10^−7^	*sinH*	Intimin-like inverse autotransporter protein SinH	2,650,403	c.399C > T	p.Pro133Pro	E8XGK6
Avian	SNP	8.8 × 10^−7^	*ilvY*	HTH-type transcriptional activator IlvY	4,116,598	c.616G > A	p.Glu206Lys	P0A2Q2
Avian	SNP	8.8 × 10^−7^	*ilvC*	Ketol-acid reductoisomerase (NADP(+))	4,117,833	c.457C > T	p.Ala153Ser	P05989
Avian	SNP	8.8 × 10^−7^	*N/A*	N/A	4,217,302	N/A	N/A	N/A
Bovine	Gene	8.6 × 10^−5^	*repE*	Replication initiation protein	N/A	N/A	N/A	P03856
Bovine	Gene	2.8 × 10^−3^	*hicB*	Antitoxin HicB	N/A	N/A	N/A	P67697
Bovine	Gene	3.7 × 10^−3^	*eptC*	Phosphoethanolamine transferase EptC	N/A	N/A	N/A	P0CB40
Bovine	SNP	1.6 × 10^−3^	N/A	N/A	294,951	N/A	N/A	N/A
Bovine	SNP	6.5 × 10^−6^	*arnD*	4-deoxy-4-formamido-L-arabinose phosphoundecaprenol deformylase ArnD	2,408,955	c.884A > C	p.Ala295Ala	O52326
Bovine	SNP	6.5 × 10^−6^	*srmB*	ATP-dependent RNA helicase SrmB	2,783,562	c.660 T > C	p.Lys220Asn	Q8ZMX7
Bovine	SNP	6.5 × 10^−6^	*aspA*	Aspartate ammonia-lyase	4,572,050	c.332C > T	p.Asn111Ile	Q7CPA1
Swine	Indel	3.3 × 10^−3^	N/A	N/A	4,816,900	N/A	N/A	N/A
Swine	SNP	4.8 × 10^−7^	*pepE*	Dipeptidase E	4,414,198	c.488G > T	p.Pro163Leu	P36936
Swine	SNP	1.7 × 10^−11^	*iroN*	TonB-dependent siderophore receptor protein	2,924,248	c.1516G > C	p.Gly506Arg	Q8ZMN0
Swine	SNP	1.7 × 10^−11^	*priA*	Primosomal protein N	4,304,871	c.689 T > C	p.Lys230Thr	Q8ZKN8
Swine	SNP	6.9 × 10^−05^	*ybeK or rihA*	Pyrimidine-specific ribonucleoside hydrolase RihA	725,582	c.912A > G	p.Ala304Ala	Q8ZQY4
Swine	SNP	2.3 × 10^−15^	*ilvY*	HTH-type transcriptional activator IlvY	4,116,897	c.317C > A	p.Leu106Gln	P0A2Q2
Fish	Gene	2.3 × 10^−8^	*dapH*	2,3,4,5-tetrahydropyridine-2,6-dicarboxylate N-acetyltransferase	N/A	N/A	N/A	Q7A2S0
Fish	Gene	3.3 × 10^−3^	*cgkA*	Kappa-carrageenase	N/A	N/A	N/A	P43478

### Metabolic pathways mainly impacted by mutations associated with animal sources (i.e. GOEA)

Based on the mutations associated by microbial GWAS (Table 3 and Additional file 8), the GO-terms retrieved by GOEA (Additional file 9) were parsed to retain the most accurate (i.e. GO-levels ≥5) and the most enriched (i.e. Bonferroni corrected *p*-values < 5.0 × 10^− 2^), as previously described [11]. This resulted in 6, 1, 0 and 2 GO-terms of interest for the avian, bovine, swine and fish sources, respectively (Table 5). These GO-terms (Table 5) were mainly related to molecular functions (i.e. 66%) and biological processes (i.e. 33%).

**Table 5:** GO-terms mainly enriched by GOEA applied on accessory genes and coregenome variants of *Salmonella enterica* subsp. *enterica* serovars associated by microbial GWAS with animal sources (i.e. avian bovine, swine and fish). The GOEA was performed with the workflow ‘fastGSEA’ based on the parent-child approach integrating hypergeometric tests and Bonferroni corrections. The GOEA input sample is a list of corresponding RefSeq identifiers of accessory genes (i.e. RefSeq from Roary) and coregenome variants (i.e. NP from SNPeff 4.1 g) associated by microbial GWAS. The input universe is a list of RefSeq identifiers of all accessory genes (i.e. RefSeq from Roary) and all core genes (i.e. NP from SNPeff 4.1 g). The highest GO-levels presenting the most accurate GO-terms (i.e. ≥ 5) and the lowest Bonferroni corrected *p*-values representing highly enriched GO-terms (i.e. < 5.0 × 10^−2^), are presented. BP, MF and CC stand for biological process, molecular function and cellular component, respectively

Animal source	Uniprotkb	Associated Mutations	GO-term identifier	GO-term	Hits	Exp. hits	GO level	Corr. *p*-value	Ontology
avian	Q55434	gene *cph2_2*	GO:0009585	red, far-red light phototransduction	1	0.01	7	1 × 10^−7^	BP
avian	Q55434	gene *cph2_2*	GO:0009584	detection of visible light	1	0.01	7	1 × 10^−7^	BP
avian	Q55434	gene *cph2_2*	GO:0009883	red or far-red light photoreceptor activity	1	0.01	5	1 × 10^−7^	MF
avian	Q9RT63	gene *recD2*	GO:0043141	ATP-dependent 5′-3′ DNA helicase activity	1	0.01	11	1 × 10^−7^	MF
avian	Q9RT63	gene *recD2*	GO:0008094	DNA-dependent ATPase activity	5	0.28	10	1 × 10^−3^	MF
avian	P0ABN5	gene *dcuA*	GO:0015740	C4-dicarboxylate transport	3	0.13	10	1 × 10^−2^	BP
bovine	Q7CPA1	SNP in *aspA*	GO:0008797	aspartate ammonia-lyase activity	1	0.01	6	1 × 10^−7^	MF
fish	Q7A2S0	gene *dapH*	GO:0047200	tetrahydrodipicolinate N-acetyltransferase activity	1	0.01	8	1 × 10^−7^	MF
fish	P43478	gene *cgkA*	GO:0033918	kappa-carrageenase activity	1	0.01	6	1 × 10^−7^	MF

## Discussion

### Restricted and unrestricted animal sources across *Salmonella*

*Salmonella* serovars might be considered as having restricted (mono-) or broad (multi-) animal sources. Here we used the Enterobase resource providing both genomic data and metadata to build a dataset to explore the relationships between genotype and adaptation to the animal sources (Fig. 1). As exemplified with *Escherichia* (only host-unrestricted lineages), *Campylobacter* (both host-restricted and -unrestricted lineages) and *Staphylococcus* (only host-restricted lineages), the lineages resulting of phylogenomic reconstructions reflect the genetic structure (i.e. patterns of mutations) established through either host-adapted lineages, physical barriers to colonization, or local clonal spreading induced by selection or genetic drift [12]. The restricted and unrestricted-host lineages can be the result of a diversity of genetic processes: neutral diversification, acquisition of a host-adaptive trait causing a genome-wide purge within the population, large recombination between strains creating a hybrid lineage or negative frequency-dependent selection induced by decreasing of fitness [12]. Our segmentation distinguishing mono- and multi-animal sources should consequently reflect a representation of clonal and panmictic serovars (Additional file 1) [43] rather than a phenomenon of adaptation to single or multiple niches. This hypothesis is supported by our ability to correct population structure considering both serovars from potential mono- and multi-animal sources as genomes of interest during microbial GWAS (Additional files 6 and 7).

### Genetic signatures of *Salmonella* adaptation to animal sources

Especially in highly recombinant bacterial genomes, phylogeographic signatures can be weakened due to dissemination around the world and genomic changes occurring within the reservoir hosts [70]. Even with a dataset of genomes highly diversified in terms of serovars (i.e. 12 clonal and 3 panmictic serovars including 13 monophyletic and 2 polyphyletic serovars), geographical origin (i.e. 26 countries, 68% from United States) and time of isolation (i.e. 25^th^ and 75^th^ percentiles: 2005–2013) origins (Additional file 3), we were able to identify genetic signatures of animal sources (Table 2, Table 4 and Additional file 8) by microbial GWAS (Fig. . 4 and Additional file 7). Host-associated genetic signatures have been previously detected for *Staphylococcus aureus* [71] and *Campylobacter* [72] which expanded into vast open livestock niches from humans [73] or pre-agriculture wild animal [73, 74]. Probably because *Salmonella enterica* subsps. *enterica* did not evolve as an obligate intracellular pathogens, we did not observe evidence of accumulation of deleterious mutations and losses of unessential functions (Fig. 2 and Table 2), that have been associated [12], or not [5], with host adaptation and restricted host transmission in other organisms. *Salmonella* genomes from human source were not included in the dataset of genomes, because it would conceal the mutations associated with the avian, bovine, swine and fish sources, but the mutations identified in the present study as associated with animal sources (Table 2 and Additional file 8) could be used as in silico or in vitro markers to identify them from human isolates in a context of source tracking for food safety [1, 2]. In this framework, we plan to develop in a near future a workflow to attribute animal sources from human samples based on the markers identified in the present study to improve the models of source attribution at the genomic scale [51], as recently proposed to prediction sources of *S.* Typhimurium by machine learning Random Forest classifier [75]. Even if the annotations of hypothetical proteins associated with animal sources have to be improved in the future (Table 2 and Additional file 8), we will discuss about the annotated mutations which have been associated with animal sources (Fig. . 3).

**Fig. 4:**
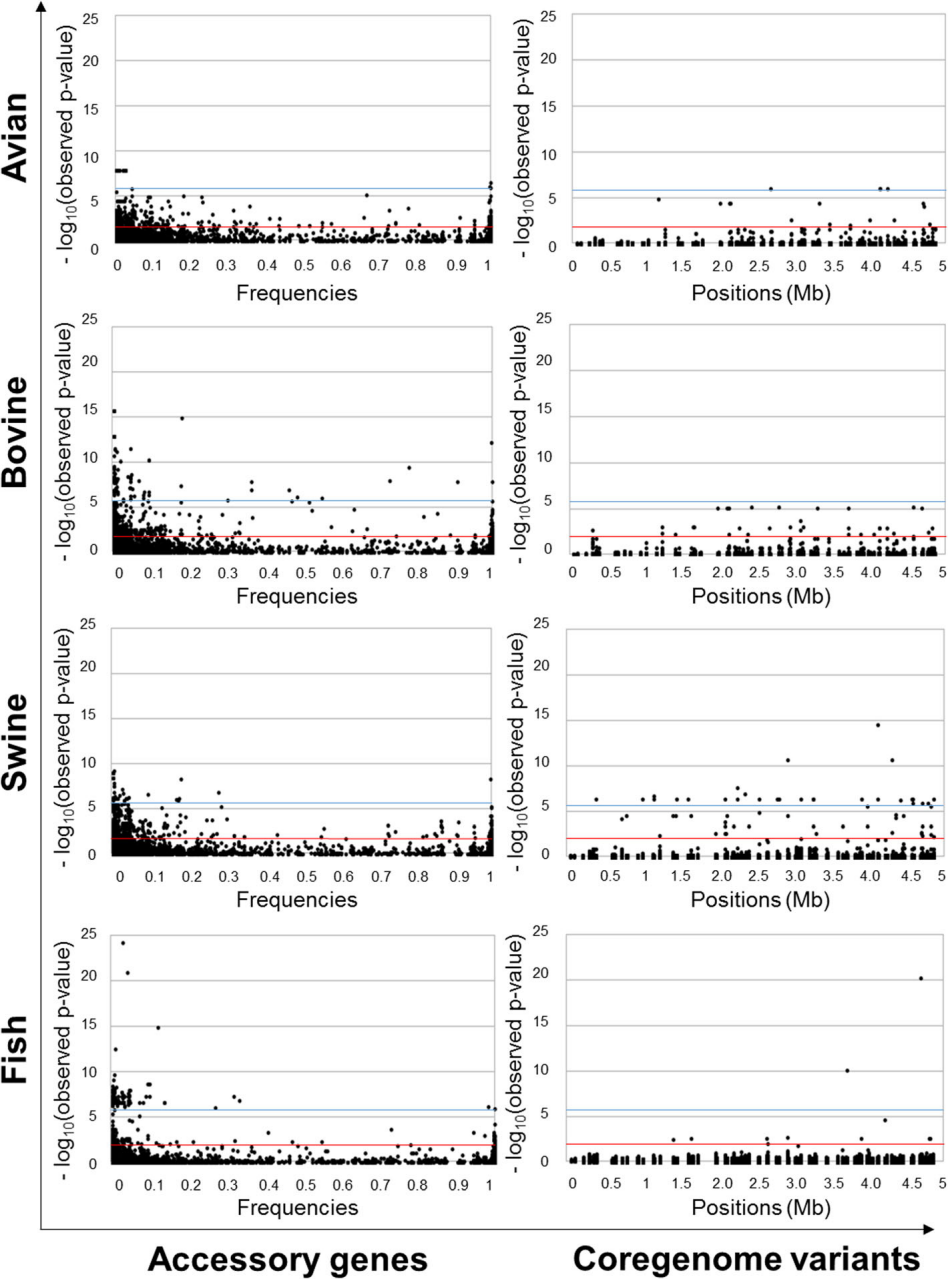
Manhattan plots from microbial GWAS aiming to detect accessory genes and coregenome variants of *Salmonella enterica* subsp. *enterica* serovars (*n* = 440) associated with avian, bovine, swine and fish sources. Strains from both potential mono- and multi-animal sources were considered as having phenotype 1 and others strains (i.e. mono-source with a different animal) as having phenotype 0. The accessory genes were sorted by frequency and the positions of coregenome variants are in accordance with the reference genome of *Salmonella* Typhimurium LT2 (NCBI NC_003197.1). The blue and red lines (− log_10_(observed *p*-values)) correspond to the genome wide significance from human GWAS and polygenicity with regard to Quantile-Quantile (QQ) plots from microbial GWAS (i.e. *p* < 1 × 10^− 2^)

### Signatures of adaptation to the avian source

The studied *Salmonella* genomes from avian sources (Additional file 3) principally come from samples found in the food chain (i.e. 92% of poultry) and may consequently be impacted by the environment and work practices of this sector. The *S.* Typhimurium mutations associated with avian source (Fig. . 3) impacted mainly the metabolism processes related to detection of visible light (GO:0009584 and GO:0009883), red and far-red light phototransduction (GO:0009585), DNA dependent activities (GO:0043141 and GO:0008094) and C4-dicarboxylate transport (GO:0015740) (Table 5).

Following the same pattern (i.e. a list of mutations observed in the same genomes), the genes *zntR2*, *cph2_2*, *merP_1* and *merP_2* are associated with avian source (Fig. 3, Table 4 and Additional file 8). The zing dependent helix-turn-helix domain (i.e. gene *zntR2*) allows binding to DNA cognate sites [76]. Never described in *Salmonella*, the cyanobacterial phytochrome Cph2 activate mobility capacity (i.e. phototaxis) towards red light (i.e. gene *cph2_2*) [77] and may be related to conditions of poultry growth were red light his frequently used to improve liveweight [78]. The mercuric transport protein periplasmic components (i.e. genes *merP_1* and *merP_2*) may also be an adaptation related to mercury exposure. DNA damage has been reported in rat and mouse fibroblasts as well as cells from Chinese hamster ovary and human cells [79]. Due to anthropogenic activities, the changes in the human chromatin is indeed known to be induced by mercury exposure of the biosphere during 500 years [80]. As recently observed in a large subantarctic avian community, the diet (i.e. crustacean, fish, squid and carrion-consumers), rather than taxonomy, is an important driver of avian mercury exposure [81]. Even if the mercury is considered as undesirable substance in animal feed by European Food Safety Authority [82], and in a context of prevention and control of contagious poultry diseases [83], the domestic avian community have been exposed to mercury through vaccination [84]. The ethyl mercury is very toxic water-soluble form of mercury developed in the 1920s to preserve vaccines, variously called Thimerosal, Merthiolate and Thiomersa. For instance, the Thimerosal contains 49% of mercury mass [85] and is a preservative used in vaccines preventing human flu (A/H1N1) [86], as well as infections of domestic poultry and other bird species with virulent Newcastle disease virus [84].

Following also the same pattern, the SNPs in genes *sinH*, *ilvY* and *ilvC* are associated with avian source (Fig. 3, Table 4 and Additional file 8). Among other delivery devices encoded in type V protein secretion systems (T5SS), the intimin-like inverse autotransporter protein SinH (i.e. synonymous SNP in *sinH*), also known as SivH, is a virulence factor involved in internal colonization of *Salmonella* [87]. Organized as a LysR protein-regulated system, the HTH-type transcriptional activator IlvY (i.e. missense SNP in *ilvC*) is the transcriptional regulator of the ketol-acid reductoisomerase NADP+ (i.e. missense SNP in *ilvC*) involved in the parallel pathway for the biosynthesis of L-isoleucine and L-valine [88], and was associated by different mutations to avian (p.Glu206Lys) and swine (p.Leu106Gln) sources (Table 4). As detailed latter concerning the diet of weaned piglets [89], the isoleucine and valine are also controlled in practical broiler formulas because these amino acids are limited in nutrition based on corn and soybean meals [90], and may consequently explain this missense SNP in *ilvC* (p.Glu206Lys) (Table 4).

Associated to different genomes of avian source, the genes *recD2* and *dcuA* are also mutations explaining this animal source. The ATP-dependent RecD-like DNA helicase (i.e. gene *recD2*) inhibits stress-induced mutations independently of effects on SOS induction in *Escherichia coli* [91]. Mediated by an anaerobic C4-dicarboxylate transporter DcuA (i.e. gene *dcuA*), *S.* Typhimurium performs a complete tricarboxylic acid cycle during colonization of the intestinal lumen to uptake and use poorly fermentable dicarboxylic acids, such as succinate, conferring a fitness advantage in competition with the native gut microbiota [92].

### Signatures of adaptation to the bovine source

All the studied *Salmonella* genomes from bovine sources are related to livestock, and like their avian counterpart, are exposed to the related environment and work practices in the food industry (Additional file 3). The mutations associated with bovine source in *S.* Cerro, *S.* Dublin and/or *S.* Meleagridis (Fig. . 3) affected the metabolism process related to aspartate ammonia-lyase activity (GO:0008797) (Table 5).

With an identical pattern, the SNP in *arnD*, as well as, the genes *arnD*, *srmB* and *aspA*, are associated with the bovine source (Fig. 3, Table 4 and Additional file 8). The 4-deoxy-4-formamido-L-arabinose phosphoundecaprenol deformylase ArnD (i.e. synonymous SNP in *arnD*) is involved in modification of LPS with arabinose and required for resistance to polymyxin and cationic antimicrobial peptides [93]. This adaptation signal may be explained by the bovine exposures to polymyxin treatments. The polymyxin and colistin (i.e. polymyxins E2 and E1) are currently last-line therapeutic options to treat infections caused by multidrug-resistant Gram-negative bacteria [94], whose residues can be detected in bovine milk and tissues [95].

The ATP-dependent RNA helicase SrmB (i.e. gene *srmB*) is a dead-box family of helicase proteins involved in ribosomal biogenesis, but his function in *Salmonella* remains to be determined [96]. The aspartate ammonia-lyase encoded by *aspA*, converts aspartate to fumarate which is reduced by a fumarate reductase into succinate [97]. As observed with enterohemorrhagic *Escherichia coli*, aspartate deamination and anaerobic fumarate respiration, may be important pathways favoring *Salmonella* adaptation to bovine gut [98].

Present in different genomes from bovine source, the genes *repE*, *hicB* and *eptC* are also associated with bovine source. The replication initiation proteins (i.e. gene *repE*) relate to incompatibility of plasmids and compete each other, with potential linked accessory genes, for replication in the bacterial host [99]. As demonstrated with *E. coli*, the antitoxin HicB (i.e. gene *hicB*) forms probably a complex with the mRNA interferase HicA which becomes active after dissociation induced by nutrient starvation and produces bacteriostatic conditions for growth of other bacterial cells [100]. The phosphoethanolamine transferase EptC (i.e. gene *eptC*) adds a phosphoethanolamine to the inner core lipooligosaccharide of *C. jejuni*, promoting recognition by a human Toll-like receptor and providing resistance to relevant mammalian and avian antimicrobial peptides [101].

### Signatures of adaptation to the swine source

The studied *Salmonella* genomes from swine sources are mainly representative of livestock of this sector (i.e. 88% of livestock) and potentially exposed to the environment and work habits of this food chain (Additional file 3). The mutations associated by microbial GWAS to different genomes of *S.* Choleraesuis, *S.* Derby and/or *S.* Rissen from swine source (Fig. 3 and Additional file 8), are not over-enriched by GOEA (Table 5) and are only constituted of core variants including an InDel in the intergenic region STM4562-*yjjU* and SNPs, as well as genes *pepE*, *iroN*, *priA*, *ybeK* and *ilvY* (Table 4). The dipeptidase E of *S.* Typhimurium (i.e. missense SNP in *pepE*) is hypothetically involved in sequestration of peptide aspartate used in synthesis of the aspartate family of amino acids [102], and the aspartate may be added in diets of piglets to improve growth performance and protect them against oxidative stress and mycotoxin infection [103]. Keeping in mind that iron availability increases the pathogenic potential of *S.* Typhimurium [104], the TonB-dependent siderophore receptor protein (i.e. missense SNP in *iroN*) is involved in iron acquisition in *S. enterica* [105]. The primosomal protein N (i.e. missence SNP in *priA*) allows restarting of stalled replication forks via its helicase activity [106] and the pyrimidine-specific ribonucleoside hydrolase RihA (i.e. synonymous SNP in *rihA*, also called *ybeK*) is involved in conversion of cytidine into cytosine [107]. Requiring more elements to consolidate the following hypothesis, this mutation associated to swine (i.e. synonymous SNP in *rihA*) may be linked to the pig specific pathway including the cytidine-5′-monophospho-N-acetylneuraminic acid hydroxylase (CMAH). This CMAH is implicated in production of carbohydrates on the surface of intestinal epithelial cells, which are considered as the primary elements interacting with microbes and viruses during foreign parasitic infection [108]. As previously emphasized, the HTH-type transcriptional activator IlvY (i.e. missense SNPs in gene *ilvY*: p.Glu206Lys in avian and p.Leu106Gln in swine) is involved in the parallel pathway for the biosynthesis of L-isoleucine and L-valine [88]. Just as the practical broiler formulas (Corzo et al. 2009), the isoleucine and valine are limited and added in the diet of weaned piglets (i.e. barley, wheat, maize and soya) [89], impacting expression of metabolisms involved in branched-chain amino acid, as well as amino acid composition of tissues [109].

### Signatures of adaptation to the fish source

The fish sources of the studied genomes may be related to environment and work habits of this food chain because the corresponding *Salmonella* samples were isolated from fresh (i.e. 28%), frozen (42%) and processed fresh (27%) fish (Additional file 3). Without annotated mutations associated with *S.* Bareilly, the mutations associated with fish source in *S.* Lexington and *S.* Brunei impacted mainly the metabolic processes involved in kappa-carrageenase (GO:0033918) and tetrahydrodipicolinate N-acetyltransferase activities (GO:0047200), respectively (Table 5). Never studied in *Salmonella*, the kappa-carrageenase (i.e. gene *cgkA*) has been described the first time in a marine bacterium *Alteromonas carrageenovora* [110] and is involved in degradation of k-carrageenan, a linear sulfated polysaccharides extracted from red edible seaweeds [111]. The 2,3,4,5-tetrahydropyridine-2,6-dicarboxylate N-acetyltransferase (i.e. gene *dapH*) is known as the first step of the L-lysine biosynthesis via diaminopimelate pathway [112] and the fish diets based on plant ingredients are deficient in lysine which is added in fish feed to improve growth [113] and liveweight [114]. These mutations associated with fish may consequently refer to adaptation induced by natural (e.g. gene *cgkA*) and artificial (e.g. gene *dapH*) diets.

## Conclusions

The strains of different serovars of the recombinant taxa *Salmonella enterica* subsp. *enterica*, evolved through clonal and panmictic lineages and adapted their genomic contents to animal sources of food chains at the accessory and coregenome scales. The major genetic and metabolic determinants of *Salmonella* adaptation to animal sources may have been driven by the natural feeding environment of the animal (e.g. k-carrageenan from red edible seaweeds for fish) and distinct livestock diets modified by human (e.g. isoleucine and valine for poultry and pig, aspartate for piglets, and lysine for fish). Environmental stimuli (e.g. red light exposure of poultry), physiological properties of the animal itself (e.g. aspartate deamination related to bovine gut adaptation), and work habits for health protection of livestock (e.g. exposure of poultry to mercury-based vaccines and exposure of bovine to polymyxin) may have also contributed to *Salmonella* adaptation underpinned by genetic and metabolic mutations associated with animal sources through the food chain.

## Methods

### Approach

We propose to decipher the adaptation to animal sources of *Salmonella* serovars. Our approach aimed at selecting 440 isolates, representative of most animal sources, sequenced using paired-end reads and recorded in a curated and synthetized subset of Enterobase (i). Secondly, accessory genes and coregenome variants (i.e. SNPs and InDels) were detected (ii). Thirdly, accessory genes and coregenome variants (i.e. SNPs and InDels) were associated with the animal sources of interest (i.e. avian, bovine, swine and fish) based on an implementation of microbial GWAS correcting for strong population structure (iii). Finally, GOEA were performed in order to decipher metabolic pathways mainly impacted by the pangenomic mutations associated with the animal sources (i.e. accessory genes and coregenome variants) (iv).

### Selection of a genome dataset (i)

With regard to metadata from Enterobase (i.e. December 2016: 83618 records), we selected 440 isolates in order to depict a high level of genomic diversity of *Salmonella enterica* subsp. *enterica* serovars, potentially related to mono- or multi-animal sources [58]. The corresponding reads were downloaded from the ENA [115]. With a homemade python script (version 2.7), the metadata from Enterobase was curated retaining complete records (i.e. BioProject, ENA ID, Host, sample Matrix, serovar, source niche, source origin, source details, country and collection years) and standardizing typos. Based on this curated subset of Enterobase (i.e. 37,747 records), the samples from environment, composite foods of the retail market and humans were not retained because they are considered as vectors of pathogen expositions and exposed susceptible consumers in the present study focusing on adaptation to animal sources (i.e. 13,635 records of considered sources). Taking into account the unbalanced distributions of serovars and sources in this curated database, indexes representative of the association levels of animal sources were calculated for each serovar and each source. These indexes ($i=\mathit{\log}\frac{\%\left(\frac{serovar}{source}\right)}{\%\left(\frac{strains}{source}\right)}$) represent the common logarithm of the number of strains per source for each specific serovar ($\%\left(\frac{serovar}{source}\right)$) divided by the number of strains per source in the full curated database ($\%\left(\frac{strains}{source}\right)$). Deviations from 0 correspond to over- or under-representation of the serovar in the source. Chi-square tests of uniformity of these indexes for each serovar allowed sorting of serovars from potential multi-animal sources (*p* > 0.02 with i ≈ 0) to potential mono-animal sources (*p* < 0.02 with i > 0 for over-represented serovars or i < 0 for under-represented serovars). Based on the curated and synthetic subset, we built a collection of 440 genomes so that its composition was genetically diversified (i.e. 15 serovars) and roughly balanced considering animal sources (i.e. mono- and multi-animal sources from 4 animal sources). More precisely, 20 genomes from each of 3 serovars from potential mono-animal sources were selected for each of the studied sources: avian, bovine, swine and fish (i.e. 240 genomes). Between 60 and 80 genomes from each of 3 serovars from potential multi-animal sources (i.e. 200 genomes) were added in order to get a roughly balanced dataset of potential mono- and multi-animal sources. The balance between mono- and multi-animal sources was deliberate and used to evaluate the impact on several confounding factors during microbial GWAS. The manual selection of isolates was performed checking the animal sources and respecting high levels of diversity concerning the geographical origins, isolation dates and BioProject accession numbers.

### Coregenome variants (ii)

The coregenome SNPs and small InDels were detected based on the variant caller HaplotypeCaller implemented in the iVARCall2 workflow [11], using *Salmonella* Typhimurium LT2 (NCBI NC_003197.1) as a reference genome, and following the best practices proposed by the Genome Analysis ToolKit [116]. More precisely, secondary alignments around small InDels were performed and duplications were excluded before variant calling analysis via local de novo assembly of haplotypes in active regions. The variants (i.e. SNPs and InDels) were flagged with SnpSift (version 4.1 g) [117] and the functional annotations of these variants were obtained with SNPeff (version 4.1 g without variants from intron, UTR-5′, UTR-3′, upstream regions, and downstream regions) [118]. As previously described, variants from homologous recombination events were detected with ClonalFrameML [13] and excluded to build set of 38,837 variants, or not to build set of 178,351 variants, with the script ‘Clonal_VCFilter’ [11]. The pseudogenomes were produced with the script ‘VCFtoPseudoGenome’ and correspond to the reference genome where the genotypes of detected variants were replaced in each genome [11].

### Accessory genome (ii)

With an in-house workflow called ARTwork, the assembly was performed based on coverage control (i.e. > 100X) with Bbmap [119], read normalization (i.e. 100X) with Bbnorm [120], control of read quality with FastQC [121], read trimming (i.e. > 20 of Quality Control) with Trimmomatic [122], de novo assembly with SPAdes [123], selection of the closely related reference genomes with MinHash among 74 reference circular genomes [124], scaffolding with MeDuSa [125], gap filling with GMcloser [126], trimming of small scaffolds (i.e. < 200 bases) with Biopython [127], as well as control of assembly quality with QUAST [128], MultiQC [129] and ggplot2 [130] graphics. Based on these draft genomes, pangenome was constructed with Roary [131] setting 95% of identity for blastp and a strict definition of the coregenome (i.e. 100% of isolates with core genes).

### Population structure (iii)

The phylogenomic reconstructions were performed based on the coregenome variants including or excluding variants from homologous recombination events (i.e. pseudogenomes from iVARCall2 [11]), as well as core genes (i.e. concatenated orthologous genes from Roary [131]). IQ-Tree [132] was applied on our datasets made up of millions of aligned sites to perform fast selections of the models of evolution based on Akaike Information Criteria (AIC) [133] and efficient tree reconstructions by maximum likelihood based on the most appropriated model of evolution [132]. More precisely, the consensus trees were produced considering all possible Nearest-Neighbor-Interchanges (NNIs) instead of only surrounded computed NNIs [132]. The search in the tree space started from a BIONJ tree [132] and an improved version [134] of the ultrafast bootstrap [135] was applied with 1000 iterations to compute boostrap support values. UFBoot convergences were checked after the IQ-Tree computation [132]. As stated in the literature, *Salmonella enterica* subsp. *indica* is the subspecies closest to subsp. *enterica* and was consequently used as an outgroup to root the tree of the subspecies *enterica* [59, 64]. Practically, this subspecies *enterica* root was identify using three isolates of the subsp. *indica* (SRR1840570, SRR1060719 and SRR1060512) and three isolates of each studied serovar. The tree distances were compared numerically with the cophenetic function of the ‘dendextend’ R package based on the Pearson, Kendall and Spearman correlations (i.e. between − 1 and + 1, referring to anti-correlated and correlated distances) [136]. The tree topologies were compared visually with the cophylo function of the ‘phytools’ R package [137]. The tree distances were also numerically compared computing the Fowlkes-Mallows index (i.e. between 0 and 1, referring to dissimilar and similar topologies, respectively) with the dendrogram function of the ‘dendextend’ R package [138], and the Robinson-Foulds index (i.e. number of different nodes between both tree) with the treedist function of the ‘phangorm’ R package [139].

### Genome wide association study (iii)

**Fig. 5:**
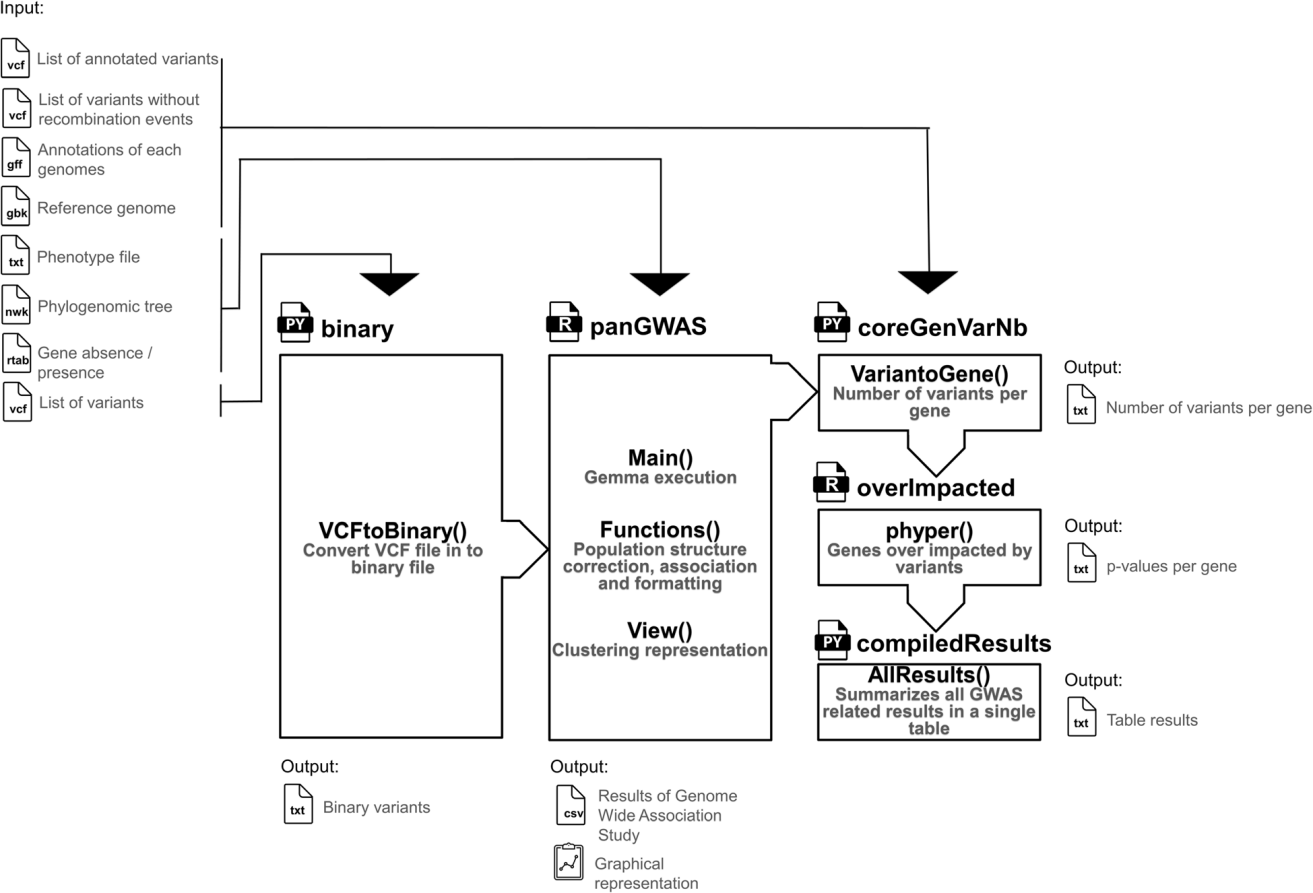
Developed scripts and published programs (i.e. black letters) with their corresponding effects (i.e. green letters) implemented in the driving script ‘microbial-GWAS’ performing microbial GWAS integrating Linear Mixed Model (LMM) for population structure correction. Based on the LMM integrated in GEMMA, the sequential workflow called ‘microbial-GWAS’ is written in R and Python 2.7. It runs successively scripts called ‘binary’, ‘panGWAS’, ‘coreGenVarNb’, ‘overImpacted’ and ‘AllResults’ in order to standardize SNPs, InDels and genes as binary data, compute Kinship matrix, fit a LMM and perform Wald tests, as well as detect coregenome variants presenting high gene densities (i.e. hotspots of variants) and high functional impacts (i.e. non-synonymous variants)

Within a range from 51 *Listeria monocytogenes* [29] to 3701 *Streptococcus pneumoniae* strains [35] and without consensus on the appropriated size of genome dataset, most of the microbial GWAS includes around 500 samples under clonal and/or panmictic status (Table 6) [43]. Contrary to human GWAS focusing on the effects of individual SNPs, microbial GWAS has also to access phenotype associations based on presence/absence of genes alongside SNPs [43]. In addition, microbial GWAS has to take into account confounding factors such as genome selection, homologous recombination events, population structure related to Linkage Disequilibrium (LD), and genome wide significance, because they can induce false positive identifications of seemingly causal mutations [43, 141]. With regard to the confounding factors (Additional file 1), we applied the developed microbial GWAS (Fig. 5) based on GEMMA [40]. This workflow was applied to 440 genomes, comparing different sizes of genome dataset, taking into account variants from homologous recombination events and checking population structure corrections. The associated mutations (i.e. Wald tests) refer to polygenicity with regard to QQ plots from microbial GWAS (i.e. *p* < 1 × 10^− 2^), and present high (i.e. > 5%) and low (i.e. < 5‰) frequencies of presence or absence (i.e. genes and alternative variants) in the studied and compared genomes, respectively.

**Table 6:** Summary of microbial GWAS. Microbial GWAS developed until now are listed comparing their workflows, mutations of interest, studied phenotypes and genome dataset

Workflow	Explicative mutations	Population structure correction	Trait	Species	Sample	Reference ^a^
bespoke	Phenotype + kmer	YES	Preferential host	*Campylobacter jejuni*	192	[30]
PhyC	Phenotype + SNP	YES	Antibiotic resistance	*Mycobacterium tuberculosis*	123	[33]
N/A	Phenotype + SNP	NO	Virulence	*Staphylococcus aureus*	90	[32]
Scoary	Gene	YES	Antibiotic resistance	*Streptococcus pneumoniae*	3085	[38]
Gemma	SNP	YES	Antimicrobial resistance	*M. tuberculosis, S. aureus, E coli, K. pneumoniae*	3144	[40]
Treewas	SNP + Gene + kmer	YES	Antimicrobial resistance	*Neisseria meningitidis*	ND	[41]
PLINK	SNP	NO	Drug resistance	*Mycobacterium tuberculosis*	123	[36]
PhyC	SNP	ND	Drug resistance	*Mycobacterium tuberculosis*	498	[39]
RoadTrips	SNP	NO	Drug Resistance	*Staphylococcus aureus*	75	[34]
PLINK	SNP	NO	Drug resistance	*Streptococcus pneumoniae*	3701	[35]
Scoary	Gene	YES	Geographical origin	*Salmonella enterica*	1327	[28]
DBGWAS	kmer	YES	Antibiotic resistance	*M. tuberculosis, S. aureus, P. aeruginosa*	1302, 992, 282	[42]
Scoary + GEMMA	Gene + SNPs	YES	Cold persistence	*Listeria monocytogenes*	51	[29]
PLINK	SNP	NO	Drug resistance	HIV	343	[140]
PLINK	SNP	NO	Viral load	HIV	1071	[31]
FaST-LMM	SNP	YES	Drug resistance	*Plasmodium falciparum*	1063	[37]

^a^ references completed from Power et al. [43]. ND stands for not determined

### Gene ontology enrichment analysis (iv)

**Fig. 6:**
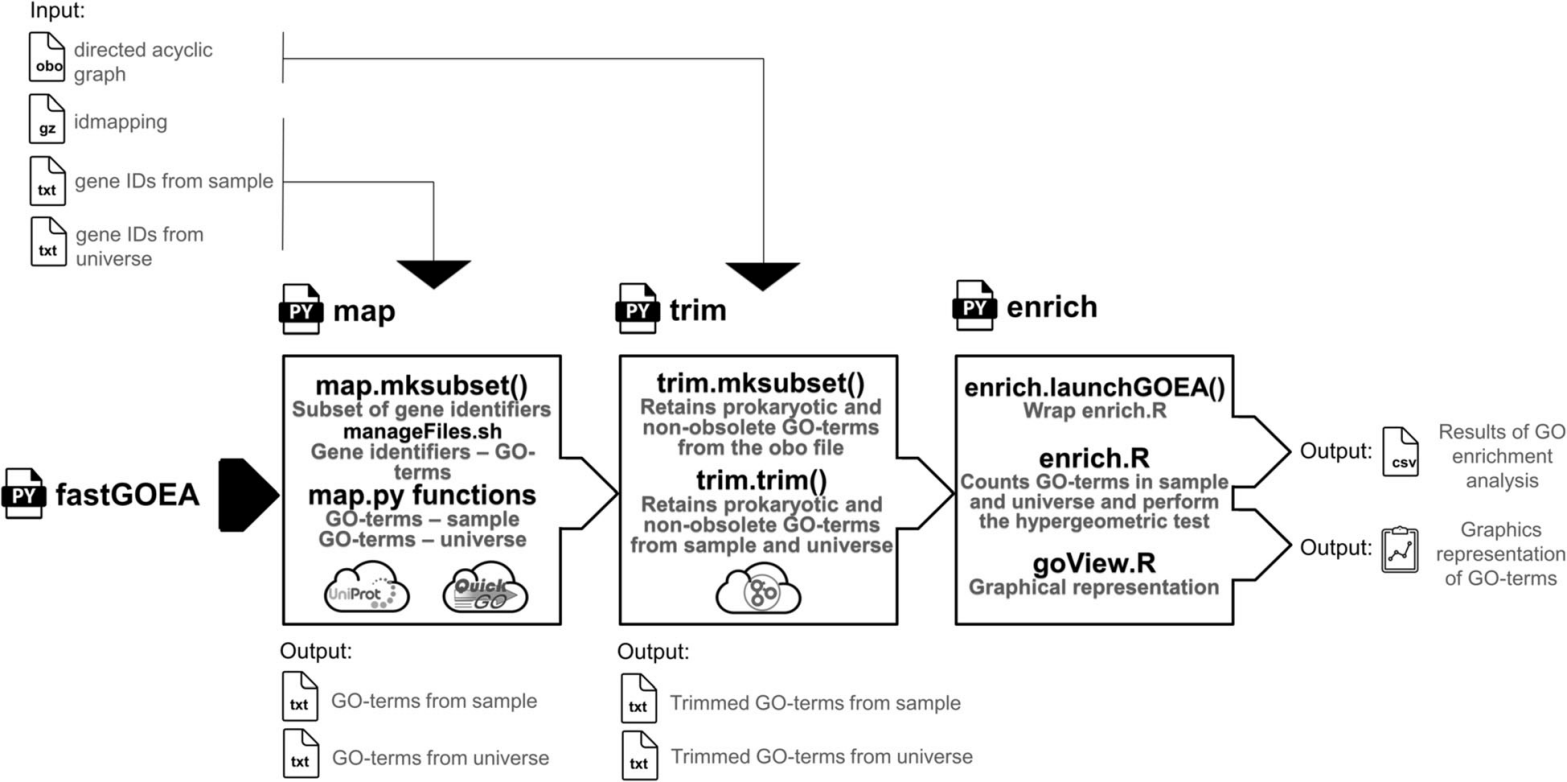
Developed scripts and published programs (i.e. black letters) with their corresponding effects (i.e. grew letters) implemented in the driving script ‘fastGOEA’ performing GOEA based on the parent-child approach integrating hypergeometric tests and Bonferroni corrections. This workflow examines the Uniprot API (i.e. slow mode) or by default a local dataset of gene identifiers (fast mode) from Uniprot (i.e. idmapping.selected.table.gz) in order to associate GO-terms to gene identifiers provided by the user as two lists referring to the sample and universe of hypergeometric tests. With regard to a directed acyclic graph (DAG) of GO-terms (i.e. go-basic.obo including eukaryotic and prokaryotic GO-terms or gosubset_prok.obo including prokaryotic GO-terms), this workflow retains prokaryotic GO-terms and avoids obsolete GO-terms before to perform GOEA. These scripts were written with Python 2.7 and implement R libraries ‘p.ajust’, ‘phyper’ and ‘ggplot2’. The whole workflow is automated and the scripts ‘trim’ and ‘enrich’ has to be performed for each GO-terms during trimming and enrichment steps, respectively

Based on our recently published workflows called ‘GetGOxML’ and ‘EveryGO’ aiming at retrieving GO-terms online from coregenome variants and perform GOEA at any node of a phylogenomic reconstruction [11], we developed an improved workflow called ‘fastGSEA’ (Fig. 6). This workflow ‘fastGSEA’ produces a fast GOEA dependently of a local Uniprot dataset of GO-terms to decrease the execution duration, and provide a complete automatic workflow applicable to many kinds of gene identifiers (i.e. 15 different gene identifiers) [56]. This workflow can also produce a slower GOEA dependently of the current version of an application programming interface provided by QuickGO (i.e. ‘Annotations’ https://www.ebi.ac.uk/QuickGO/api/index.html). The driver script ‘fastGSEA’ is written in Python (version 2.7) and uses as input a dataset of gene identifiers (i.e. idmapping.selected.table.gz; current release from Uniprot: ftp://ftp.uniprot.org/). It requires also two lists of gene identifiers from the sample of interest and universe, as well as a file representing the DAG of GO-terms (i.e. go-basic.obo including eukaryotic and prokaryotic GO-terms or gosubset_prok.obo including only prokaryotic GO-terms). The first step of the workflow ‘fastGSEA’ aims at selecting from the dataset ‘idmapping’, a subset of gene identifiers linking the gene identifiers provided by the user, corresponding Uniprot identifiers and associated GO-terms from the sample and universe lists locally (i.e. based on GO-terms from the subset) or online (i.e. based on Uniprot identifiers from the subset). Secondly, the workflow uses the DAG of GO-terms to retain prokaryotic GO-terms and avoid obsolete GO-terms. The third step of the workflow tests the hypergeometric distributions of GO-terms (i.e. ‘phyper’ R function) [142] and corrects the produced *p*-values based on the Bonferroni correction (‘p.ajust’ R function) [143]. In parallel to a file centralizing the results (i.e. GO-terms, number of hits, GO levels, *p*-values, ontology), the workflow ‘fastGSEA’ produces finally a graphical representation of the GOEA with the plotting system ggplot2 [130].

## Supplementary information

**Additional file 1.** Confounding factors of microbial GWAS. The confounding factors of microbial GWAS include the selection of genomes, homologous recombination events, population structure, as well as genome wide significance.

**Additional file 2. **Distribution of source niches and source types of *Salmonella enterica* subsp. *enterica* serovars from Enterobase among full records in terms of read identifier, serovar, source niche, source niche, source detail, BioProject accession number of the European Nucleotide Archive (ENA), date of isolation and country of isolation. Due to typos and missing information, the metadata of Enterobase was downloaded on December 2016 (83,618 records), then curated and synthesized (i.e. 37,747 records) with a homemade python script (version 2.7). The present study focusing on adaptation to animal sources (*n* = 13,635 records) does not include isolates from composite foods of the retail market and humans, which are considered as vectors of pathogen expositions and exposed susceptible consumers, respectively.

**Additional file 3. **Samples of *Salmonella enterica* subsp. *enterica* serovars studied in the present study (*n* = 440). The read identifier, serovar, source niche, source detail, BioProject accession number of the European Nucleotide Archive (ENA), date of isolation and country of isolation were retrieved from Enterobase on December 2016. ND stands for not determined and corresponds to three samples added to reach a balanced dataset of genomes.

**Additional file 4. **Boxplots (median, 25^th^ percentile, 75^th^ percentile, minimum and maximum) of depth (A) and breadth (B) coverages, numbers of scaffolds higher than 1000 bp (C), NG50 (D), genome fractions (E), and number of genes resulting of pangenome extraction (F) of *Salmonella enterica* subsp. *enterica* serovars (*n* = 440). Assembly, variant calling analysis, computing of metrics, and pangenome analysis were performed with ARTWork, iVARCall2, Quast-MultiQC and Roary, respectively. *Salmonella* Typhimurium LT2 (NCBI NC_003197.1) was used as the reference genome for mapping during variant calling analysis. Black dots represent the sizes of the closely related reference genomes selected among 74 reference-circularized genomes based on MinHash distances.

**Additional file 5. **Topology differences of phylogenomic trees of *Salmonella enterica* subsp. *enterica* serovars (*n* = 440). The phylogenomic trees were reconstructed by maximum likelihood selecting the most appropriated models of evolution and checking ultrafast bootstrap convergences (i.e. IQ-Tree). The compared approaches ‘variants’ and ‘genes’ correspond to phylogenomic reconstructions based on pseudogenomes from variant calling analysis (i.e. iVARCall2) including (A) or excluding (B) recombination events (i.e. ClonalFrameML), and concatenated orthologous genes (C) from pangenome analysis (i.e. Roary), respectively. These graphical representations were produced with the cophylo function of the ‘phytools’ R package. Most of the branches of the trees (i.e. 85, 55 and 77% for approaches A, B and C, respectively) are supported by bootstrap values higher than 90% and the corresponding newick files are accessible under request.

**Additional file 6. **Quantile-Quantile (QQ) plots from microbial GWAS aiming to identify polygenicity during associations of accessory genes and coregenome variants including homologous recombination events of *Salmonella enterica* subsp. *enterica* serovars (*n* = 440) with avian (A), bovine (B), swine (C) and fish (D) sources. The samples were assigned to potential mono- and multi-animal sources based on a curated and synthetic version of Enterobase. The absence of GEMMA convergence is represented by a cross. The red line (i.e. - log_10_(observed *p*-values) = - log_10_(expected *p*-values)) corresponds to the reference line reflecting the level of population structure correction.

**Additional file 7. **Quantile-Quantile (QQ) plots from microbial GWAS aiming to identify polygenicity during associations of accessory genes and coregenome variants excluding homologous recombination events of *Salmonella enterica* subsp. *enterica* serovars (*n* = 440) with avian (A), bovine (B), swine (C) and fish (D) sources. The samples were assigned to potential mono- and multi-animal sources based on a curated and synthetic version of Enterobase. The absence of GEMMA convergence is represented by a cross. The red line (i.e. - log_10_(observed *p*-values) = - log_10_(expected *p*-values)) corresponds to the reference line reflecting the level of population structure correction.

**Additional file 8. **Microbial GWAS results aiming to associate accessory genes and coregenome variants of *Salmonella enterica* subsp. *enterica* serovars (*n* = 440) with animal sources (i.e. avian bovine, swine and fish). The microbial GWAS was performed with the workflow ‘microbial-GWAS’ based on Linear Mixed Model (LMM) for population structure correction and Wald tests for association. The genome dataset includes both genomes assigned to potential mono- and multi-animal sources based on a curated and synthesized version of Enterobase. The associated mutations (i.e. Wald tests) refer to polygenicity with regard to Quantile-Quantile﻿ (QQ) plots from microbial GWAS (i.e. *p* < 1 × 10^− 2^) and present high (i.e. > 5%) and low (i.e. < 5‰) frequencies of presence or absence (i.e. genes and alternative variants) in the studied and compared genomes, respectively.

**Additional file 9. **GO-terms enriched by GOEA applied on accessory genes and coregenome variants of *Salmonella enterica* subsp. *enterica* serovars (*n* = 440) associated with animal sources (i.e. avian bovine, swine and fish). The GOEA was performed with the workflow ‘fastGSEA’ based on the parent-child approach integrating hypergeometric tests and Bonferroni corrections. The GOEA input sample is a list of corresponding RefSeq identifiers of accessory genes (i.e. RefSeq from Roary) and coregenome variants (i.e. NP from SNPeff 4.1 g) associated by microbial GWAS. The input universe is a list of RefSeq identifiers of all accessory genes (i.e. RefSeq from Roary) and all core genes (i.e. NP from SNPeff 4.1 g). BP, MF and CC stand for Biological Process, Molecular Function and Cellular Component, respectively.

## Data Availability

BioProjets and sequencing reads analysed during this study are included in Additional file 3. All data generated or analysed during this study are included in this published article and its Additional files. The scripts of the developed workflow called ‘microbial-GWAS’ can be found in the following GitHub repository: https://github.com/VilaNovaMeryl. The scripts of the developed workflow called ‘fastGSEA’ can be found in the following GitHub repository: https://github.com/KDurimel/DNAlogy/tree/master/FAST_GOEA.

## Abbreviations

AIC: Akaike Information Criteria
BP: Biological Process
CC: Cellular Component
CMAH: Cytidine-5′-monophospho-N-acetylneuraminic acid hydroxylase
EFSA: European Food Safety Authority
ENA: European Nucleotide Archive
GOEA: Gene Ontology Enrichment Analysis
GWAS: Genome Wide Association Study
HGT: Horizontal Gene Transfers
InDels: Small insertions/deletions
LD: Linkage Disequilibrium
MF: Molecular Function
MGEs: Mobile Genetic Elements
NNIs: Nearest-Neighbor-Interchanges
QQ plots: Quantile-Quantile plots
SPIs: *Salmonella* Pathogenicity Islands
T3SS: Type III secretion systems
T5SS: Type V protein secretion systems
