# Supplementary material for: Genetic and metabolic signatures of Salmonella enterica subsp. enterica associated with animal sources at the pangenomic scale
Source: BMC Genomics. 2019 Nov 6;20:814. doi: 10.1186/s12864-019-6188-x (PMC6836353; doi:10.1186/s12864-019-6188-x)
Supplement: Supplementary file 1 — Additional file 1. Confounding factors of microbial GWAS. The confounding factors of microbial GWAS include the selection of genomes, homologous recombination events, population structure, as well as genome wide significance. [file 12864_2019_6188_MOESM1_ESM.pdf]

Genome selection (i.e. establishing of the dataset of genomes)

Human GWAS is used to recruiting samples from ethnically homogenous clusters in order to avoid subtle confounding from population structure induced by an excess of samples from one ethnic cluster. This approach is not feasible with bacterial lineages presenting stronger population stratification caused by co-existing clonal and panmictic stats [43]. In order to avoid false positives and to accurately estimate the effect size of the mutation, the gold standard for reporting associations from human GWAS is cohorts of independent samples [44], but microbial GWAS are less reliant on cohort replication because associated mutations can be tested *in vitro* [43].

Recombination (i.e. consideration of the homologous recombination evens)

Correlations between genetic mutations in close proximity, also called linkage disequilibrium (LD), persist because of co-inheritance over generations of genomic segments [45]. In opposite to multiple cross-overs along the whole chromosome, the homologous recombination events correspond to the replacements of short sequence blocks [43] and may conceal the detection of causal variants by microbial GWAS [46]. Indeed, the recombination events break down the long-range LD into short-range LD where potentially causal variants are confounded with variants from recombination events [47]. Nevertheless, there is no consensus concerning the fact that variants located in and originating from homologous recombination events has to be included, or not, from microbial GWAS.

Population structure (i.e. differences between stratification and polygenicity)

Owing to the microbial population structure ranging from purely clonal to nearly panmictic [43], the causal variants may be common across genomes under strong selection and inherit rapidly and successfully into specific lineages implicated in a phenotype of interest (i.e. clonal genomes with few recombination events). In reverse [43], the variants causing this phenotype of interest may not be inherited from the most common ancestor and be present in almost all the known lineages under weak selection (i.e. panmictic genomes with many recombination evens). In contrast to typical human GWAS regression methods, these different features of microbial population structure led the microbial GWAS to apply mixed models in order to take into account relatedness [48], also called lineage effects [40], or

identify signals of selection based on phylogenetic structure [33]. According to Power *et al.*, disentangling the effects of a single mutation from those related to lineage is complex for bacteria [43] but feasible as successfully implemented with mixed models accounting for relatedness by Earle *et al*. [40]. Usually, the quantile-quantile (QQ) plot is used to compare the distribution of negative common logarithm of the p-values observed in the study (i.e. y-axis) to the expected distribution under the null hypothesis (x-axis) according to a reference line (i.e. $y = x$) reflecting the level of population structure correction. More precisely, while a systematically inflated $-\log_{10}$(observed p-values) for all mutations reflects strong population stratification in a QQ plot, their inflation for only high $-\log_{10}$(observed p-values) reflects polygenicity [43]. Although polygenic patterns of QQ plots emphasize successful qualitatively corrections of population structure during human or microbial GWAS, we propose to use them to refine the more appropriate genome wide significance for microbial GWAS.

Genome wide significance (i.e. p-values of association)

Because of independent multiple testing, human GWAS proposed stringent genome wide significance cut-off until $p < 5x10^{-8}$ (i.e. approximately equal to the Bonferroni correction in earlier GWAS) [50], rather than a usual statistically significant value of $p < 5x10^{-2}$ retaining tens of thousands associated mutations from hundreds of thousands analyzed mutations [43]. The GWAS associations are visualized with a Manhattan plot representing the negative common logarithm of the p-values of association (i.e. y-axis) of each mutation (i.e. x-axis) according to a reference line (i.e. $y =$ constant values) reflecting genome wide significance values commonly higher than $-\log_{10}$(p-value) = 5 or 6 in human GWAS (i.e. excluding mutations that are out of Hardy-Weinberg equilibrium). On the other hand, it must be emphasized that a single extremely significant mutation is often interpreted as a genotyping error because of the expected LD in both human and microbes [43]. Because there is no consensus about genome wide significance for microbial GWAS, we propose to define it based on polygenic patterns from the related QQ plots.
