# Supplementary figures and images for: Genetic and metabolic signatures of Salmonella enterica subsp. enterica associated with animal sources at the pangenomic scale
Source: BMC Genomics. 2019 Nov 6;20:814. doi: 10.1186/s12864-019-6188-x (PMC6836353; doi:10.1186/s12864-019-6188-x)

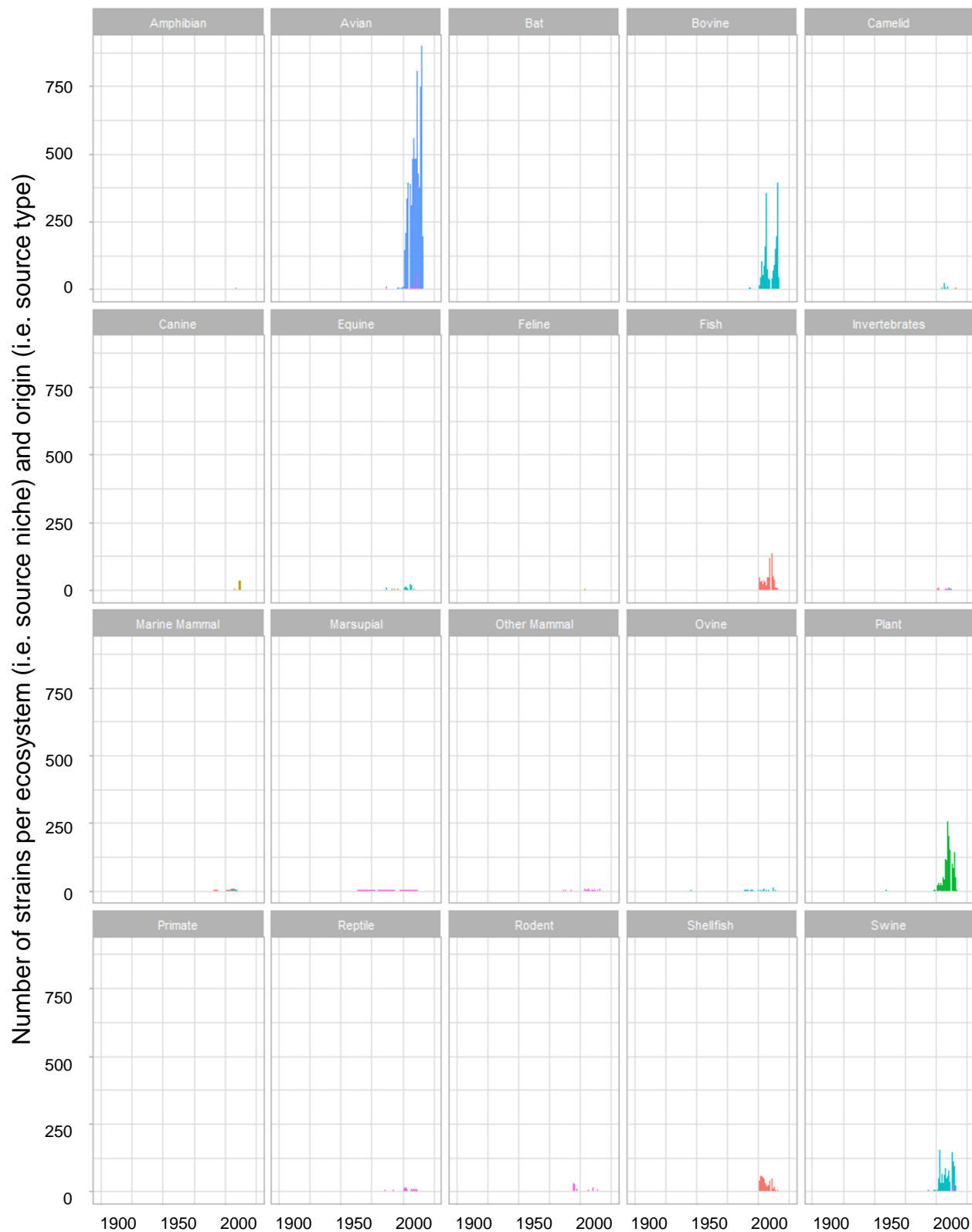

Year of strain isolation (13 635 strains and 277 serovars)

Niche

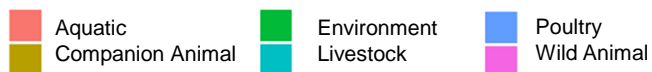

Supplement: Supplementary file 2 — Additional file 2. Distribution of source niches and source types of Salmonella enterica subsp. enterica serovars from Enterobase among full records in terms of read identifier, serovar, source niche, source niche, source detail, BioProject accession number of the European Nucleotide Archive (ENA), date of isolation and country of isolation. Due to typos and missing information, the metadata of Enterobase was downloaded on December 2016 (83,618 records), then curated and synthesized (i.e. 37,747 records) with a homemade python script (version 2.7). The present study focusing on adaptation to animal sources (n = 13,635 records) does not include isolates from composite foods of the retail market and humans, which are considered as vectors of pathogen expositions and exposed susceptible consumers, respectively. [file 12864_2019_6188_MOESM2_ESM.pdf]

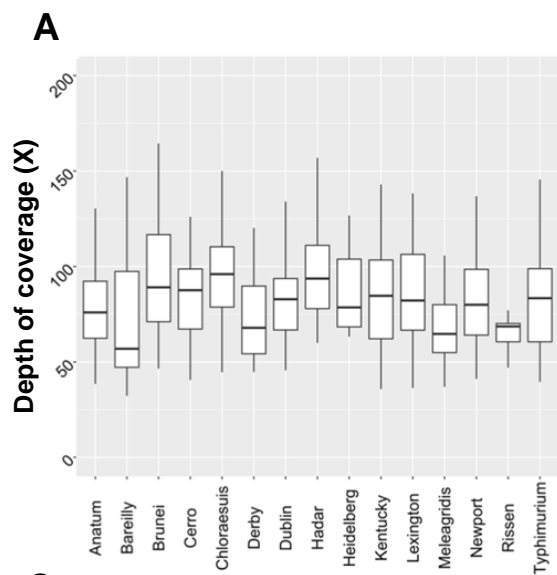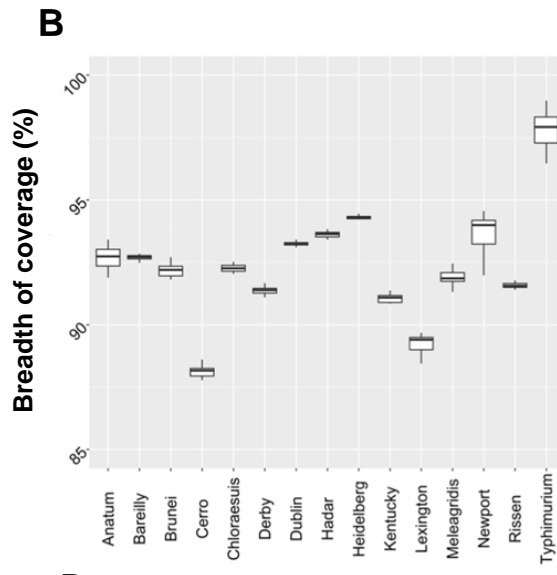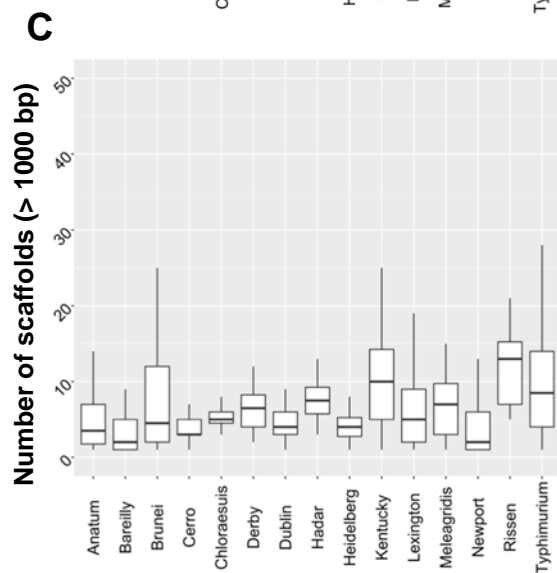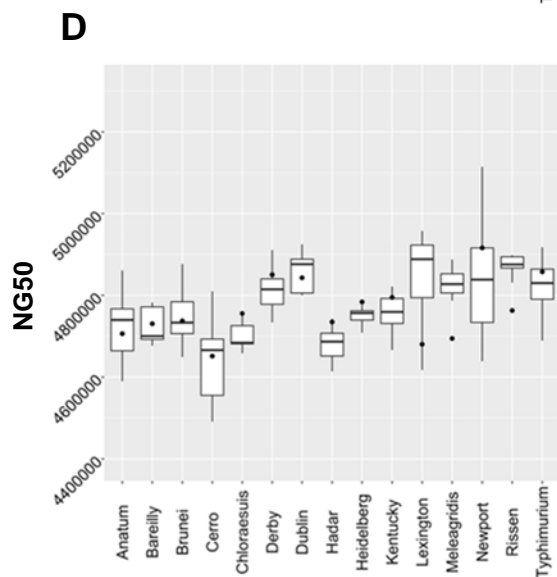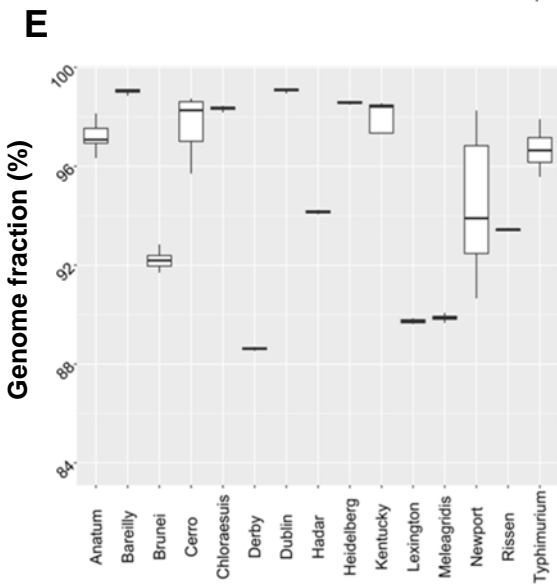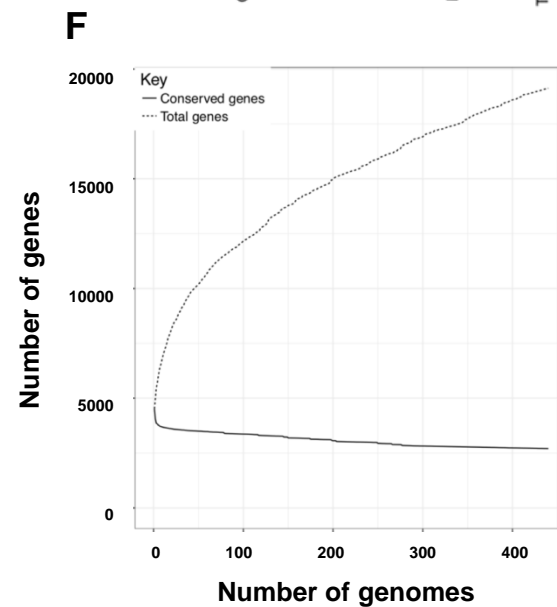

Supplement: Supplementary file 4 — Additional file 4. Boxplots (median, 25th percentile, 75th percentile, minimum and maximum) of depth (A) and breadth (B) coverages, numbers of scaffolds higher than 1000 bp (C), NG50 (D), genome fractions (E), and number of genes resulting of pangenome extraction (F) of Salmonella enterica subsp. enterica serovars (n = 440). Assembly, variant calling analysis, computing of metrics, and pangenome analysis were performed with ARTWork, iVARCall2, Quast-MultiQC and Roary, respectively. Salmonella Typhimurium LT2 (NCBI NC_003197.1) was used as the reference genome for mapping during variant calling analysis. Black dots represent the sizes of the closely related reference genomes selected among 74 reference-circularized genomes based on MinHash distances. [file 12864_2019_6188_MOESM4_ESM.pdf]

A

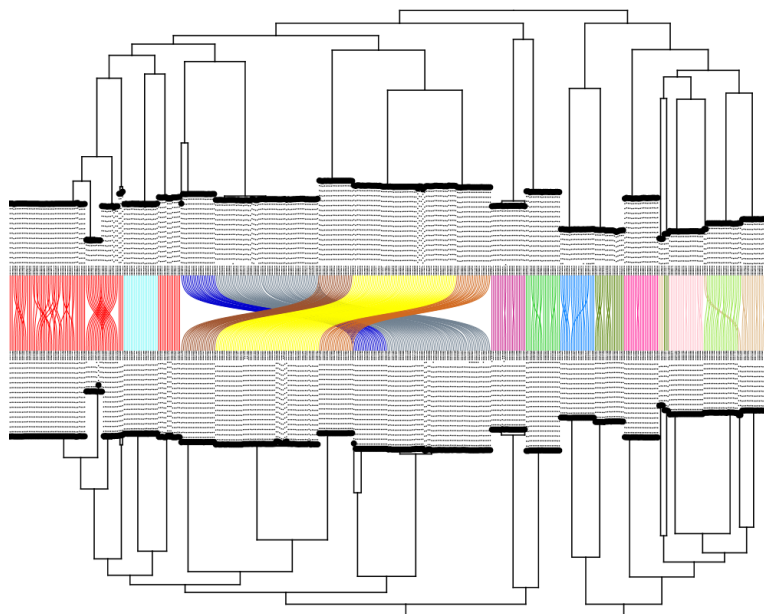

B

A

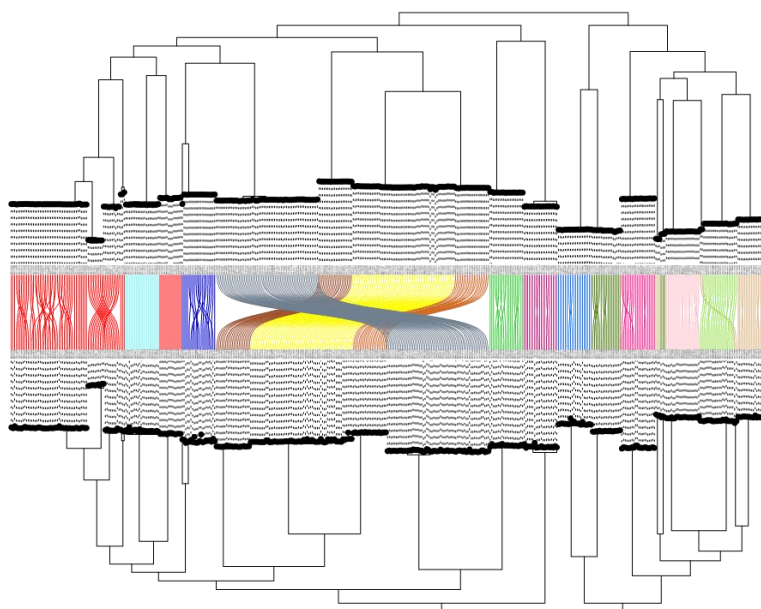

C

B

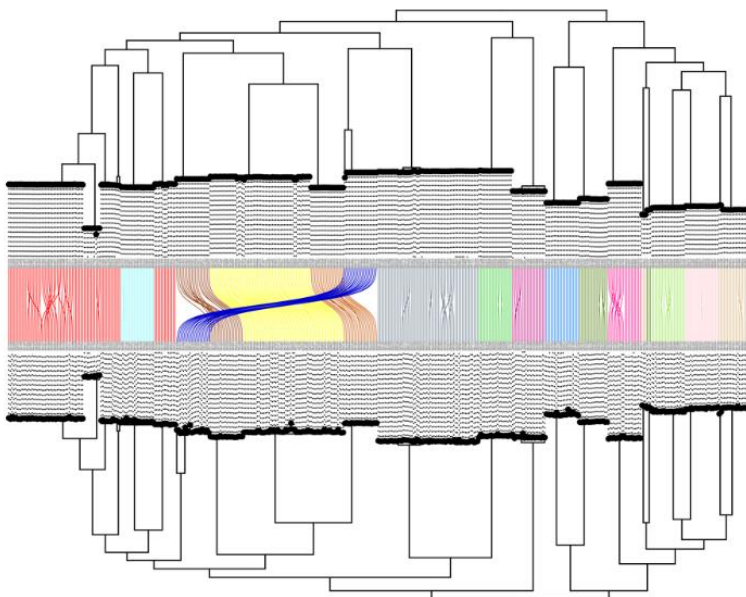

C

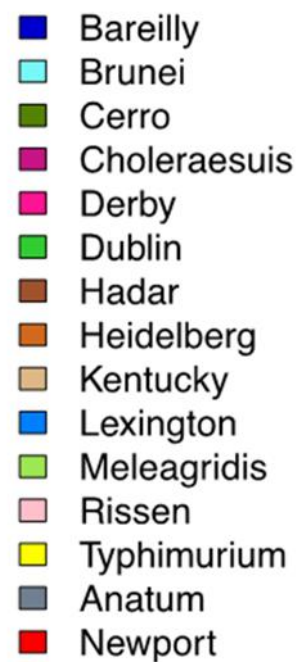

Supplement: Supplementary file 5 — Additional file 5. Topology differences of phylogenomic trees of Salmonella enterica subsp. enterica serovars (n = 440). The phylogenomic trees were reconstructed by maximum likelihood selecting the most appropriated models of evolution and checking ultrafast bootstrap convergences (i.e. IQ-Tree). The compared approaches ‘variants’ and ‘genes’ correspond to phylogenomic reconstructions based on pseudogenomes from variant calling analysis (i.e. iVARCall2) including (A) or excluding (B) recombination events (i.e. ClonalFrameML), and concatenated orthologous genes (C) from pangenome analysis (i.e. Roary), respectively. These graphical representations were produced with the cophylo function of the ‘phytools’ R package. Most of the branches of the trees (i.e. 85, 55 and 77% for approaches A, B and C, respectively) are supported by bootstrap values higher than 90% and the corresponding newick files are accessible under request. [file 12864_2019_6188_MOESM5_ESM.pdf]

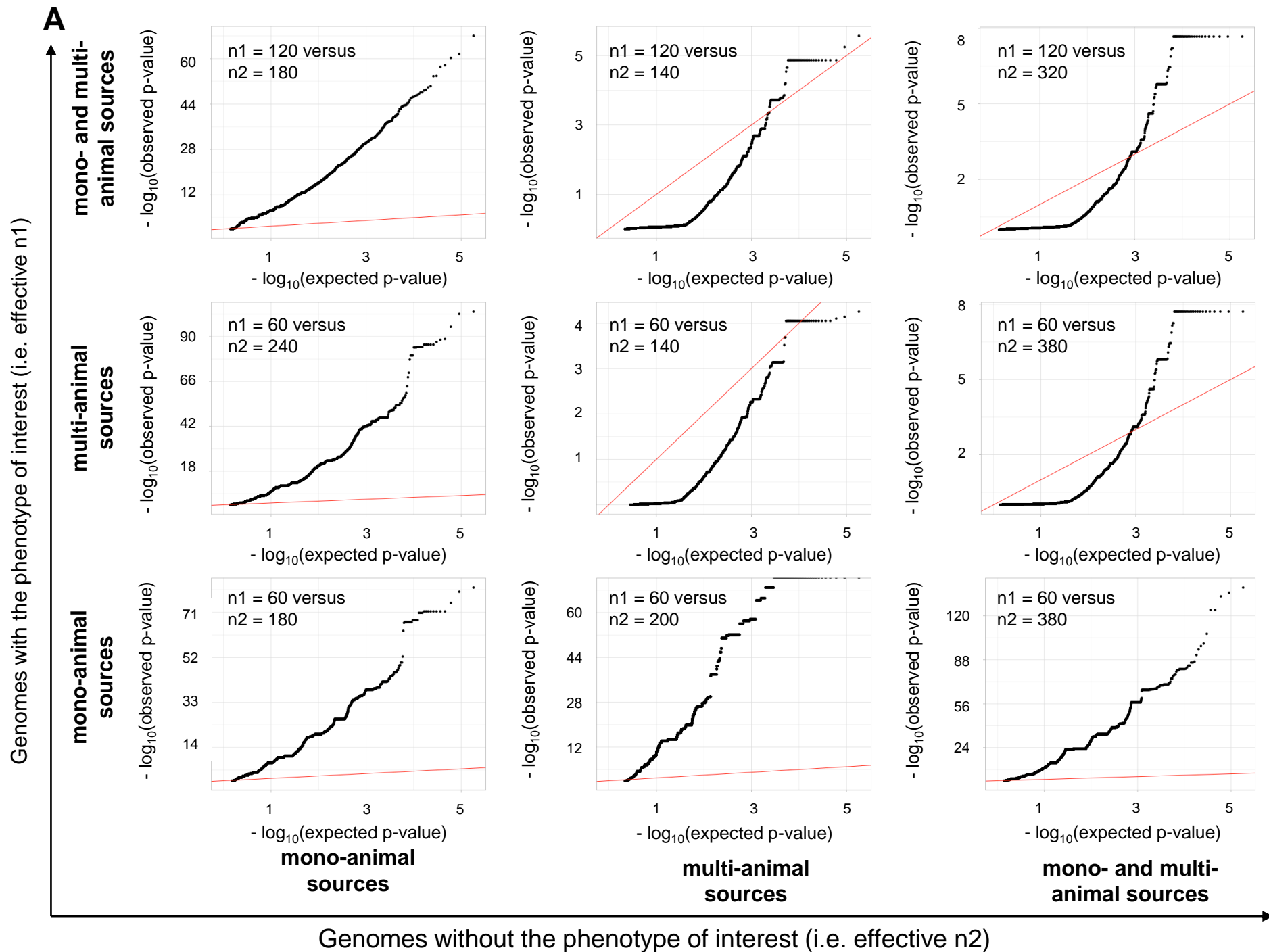

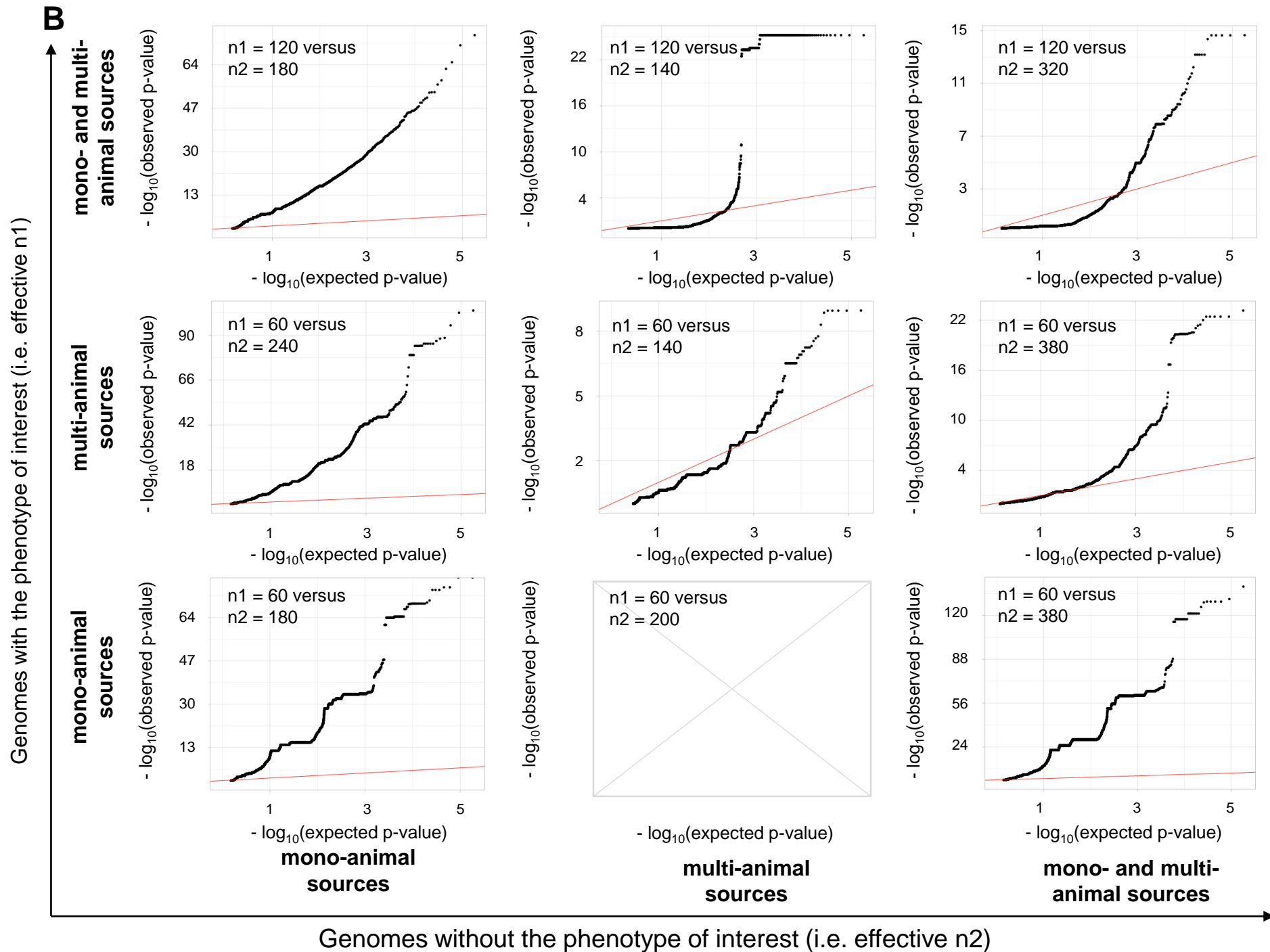

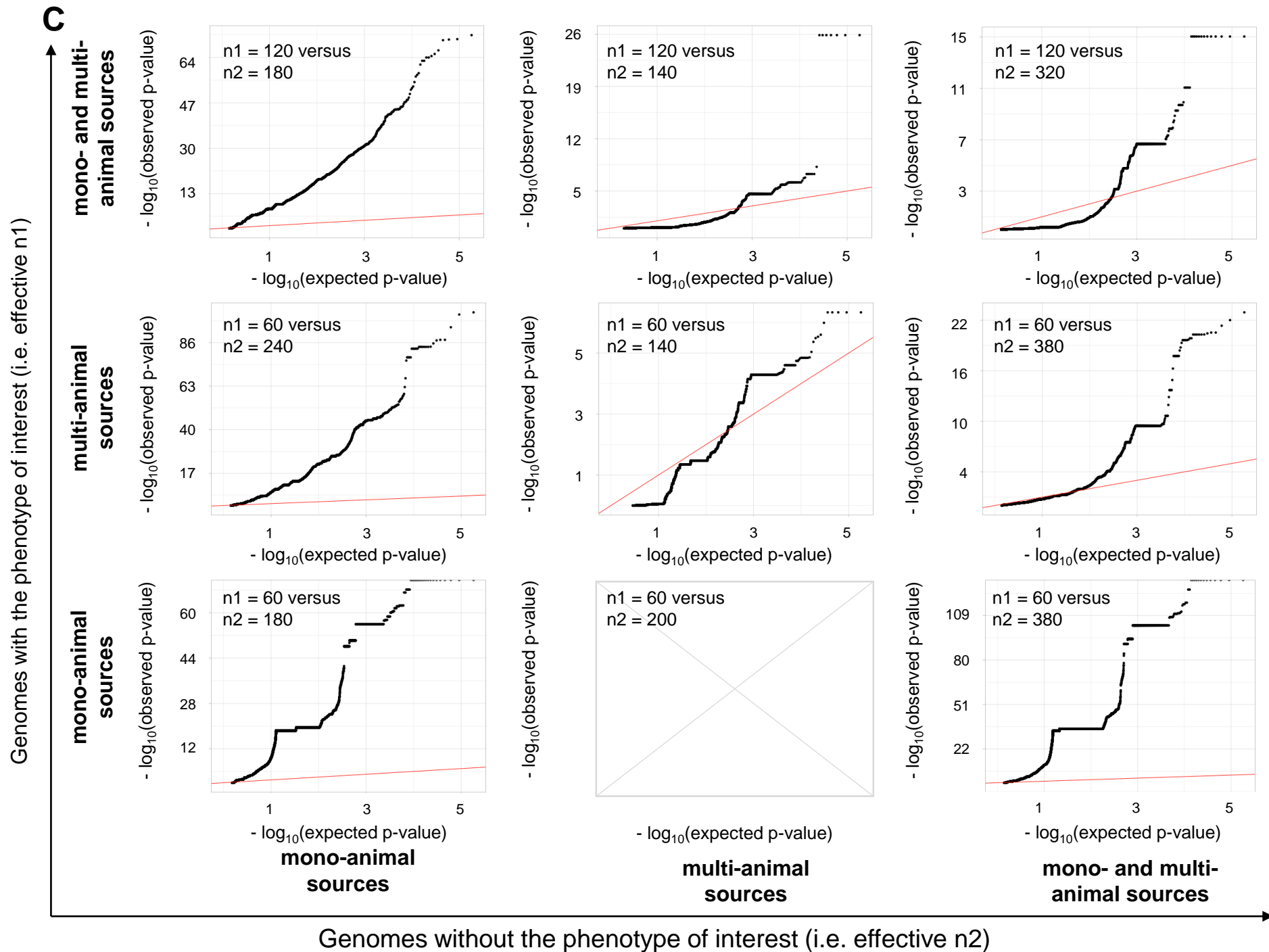

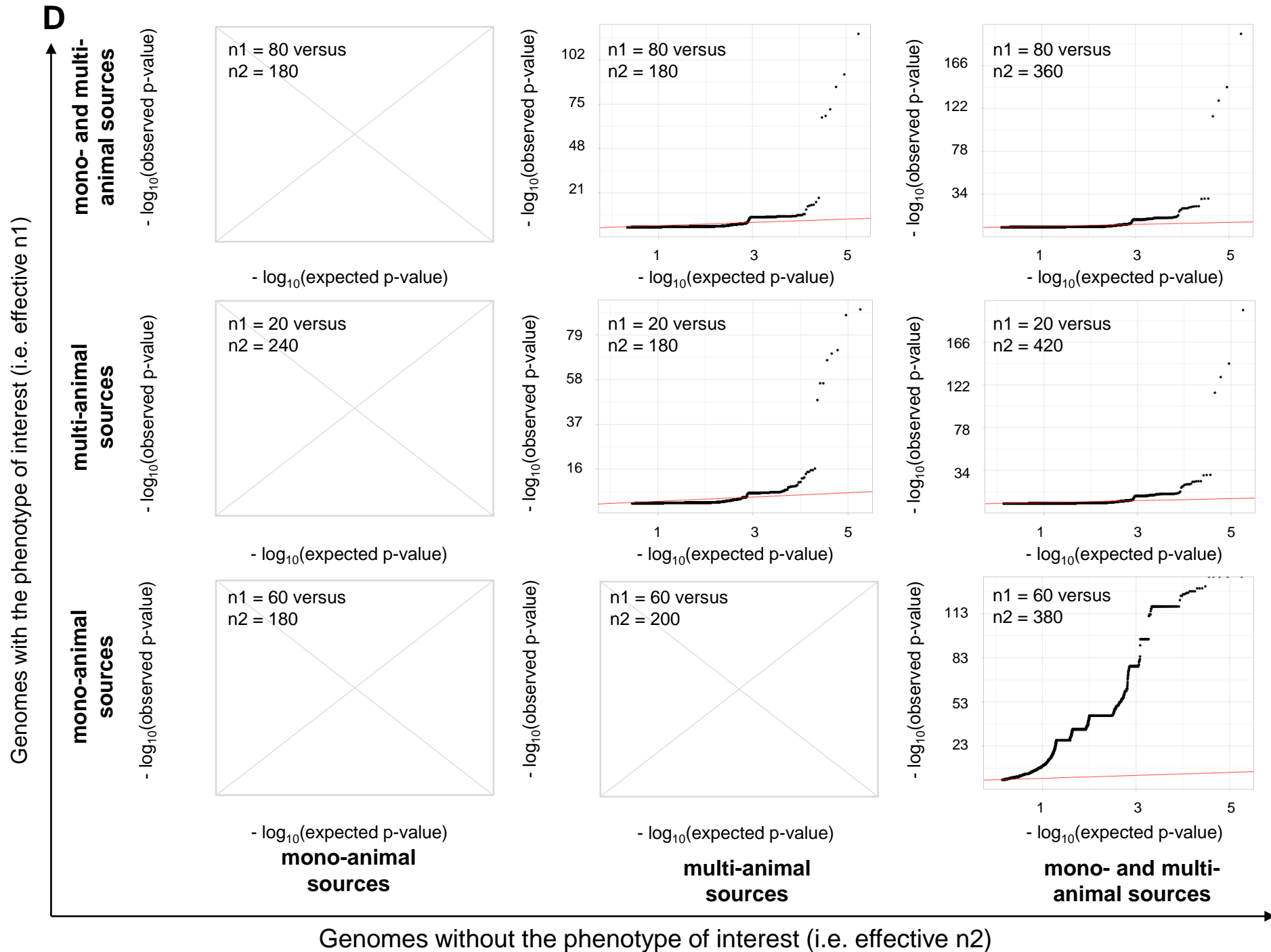

Supplement: Supplementary file 6 — Additional file 6. Quantile-Quantile (QQ) plots from microbial GWAS aiming to identify polygenicity during associations of accessory genes and coregenome variants including homologous recombination events of Salmonella enterica subsp. enterica serovars (n = 440) with avian (A), bovine (B), swine (C) and fish (D) sources. The samples were assigned to potential mono- and multi-animal sources based on a curated and synthetic version of Enterobase. The absence of GEMMA convergence is represented by a cross. The red line (i.e. - log10(observed p-values) = - log10(expected p-values)) corresponds to the reference line reflecting the level of population structure correction. [file 12864_2019_6188_MOESM6_ESM.pdf]

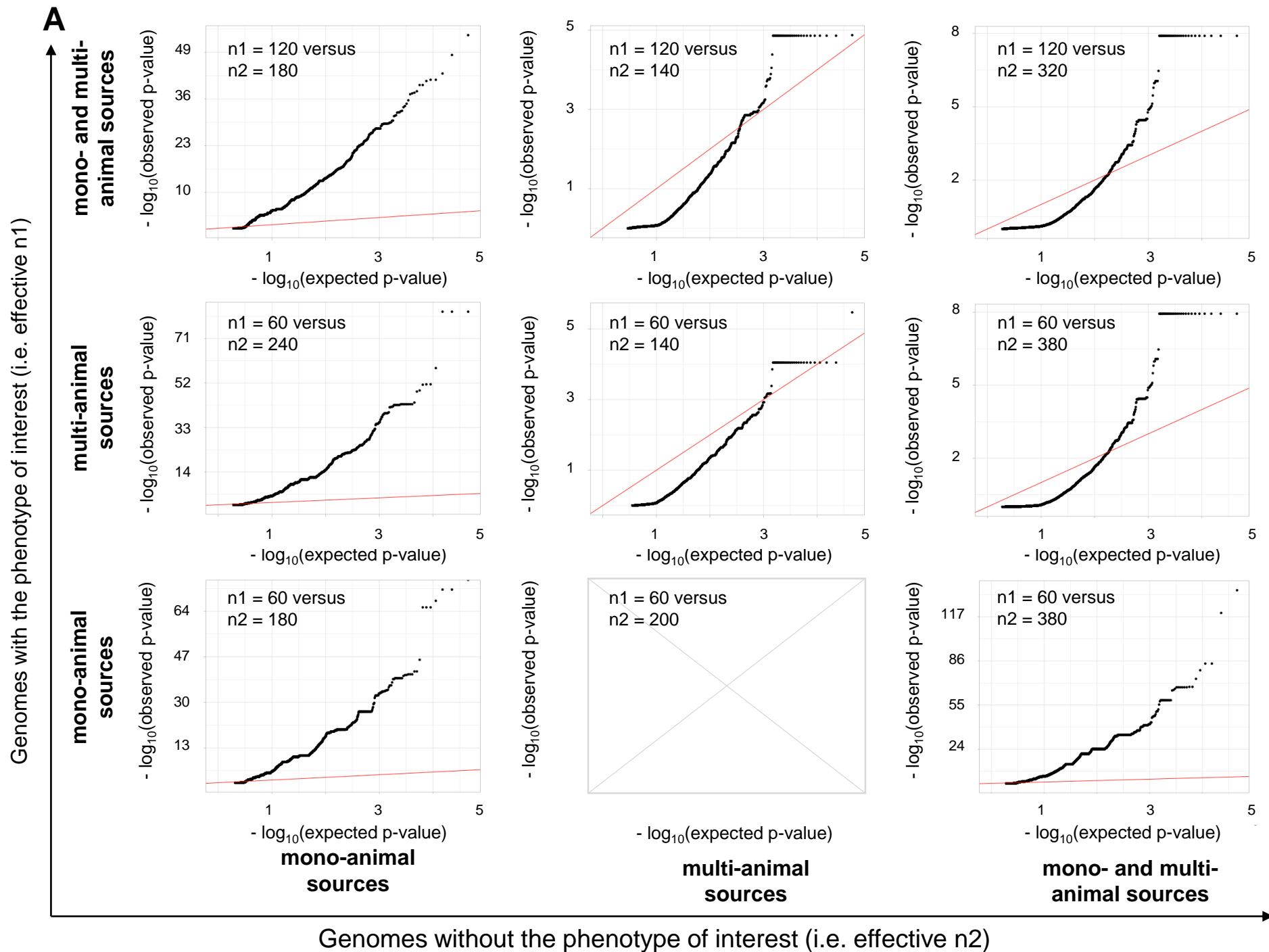

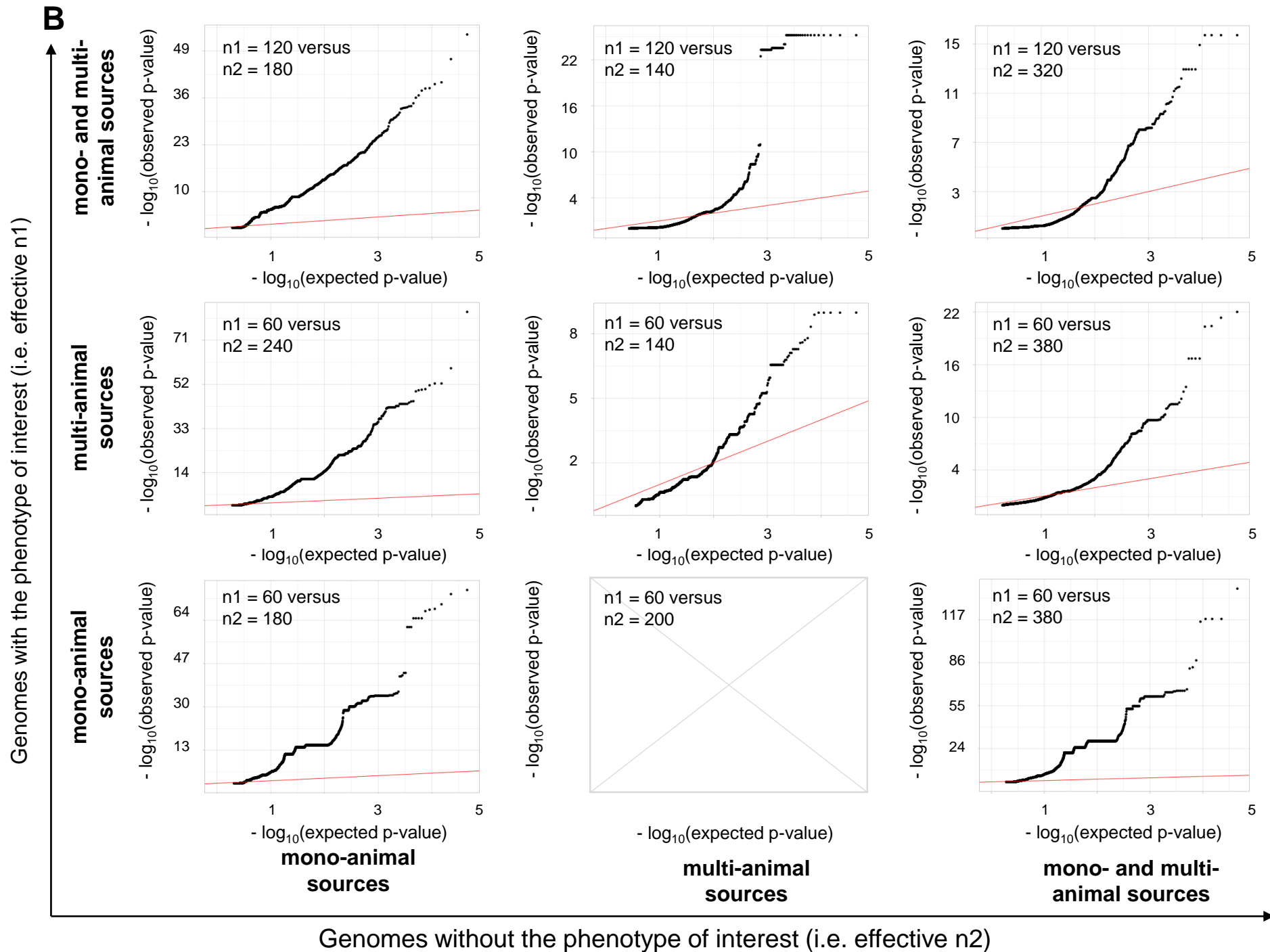

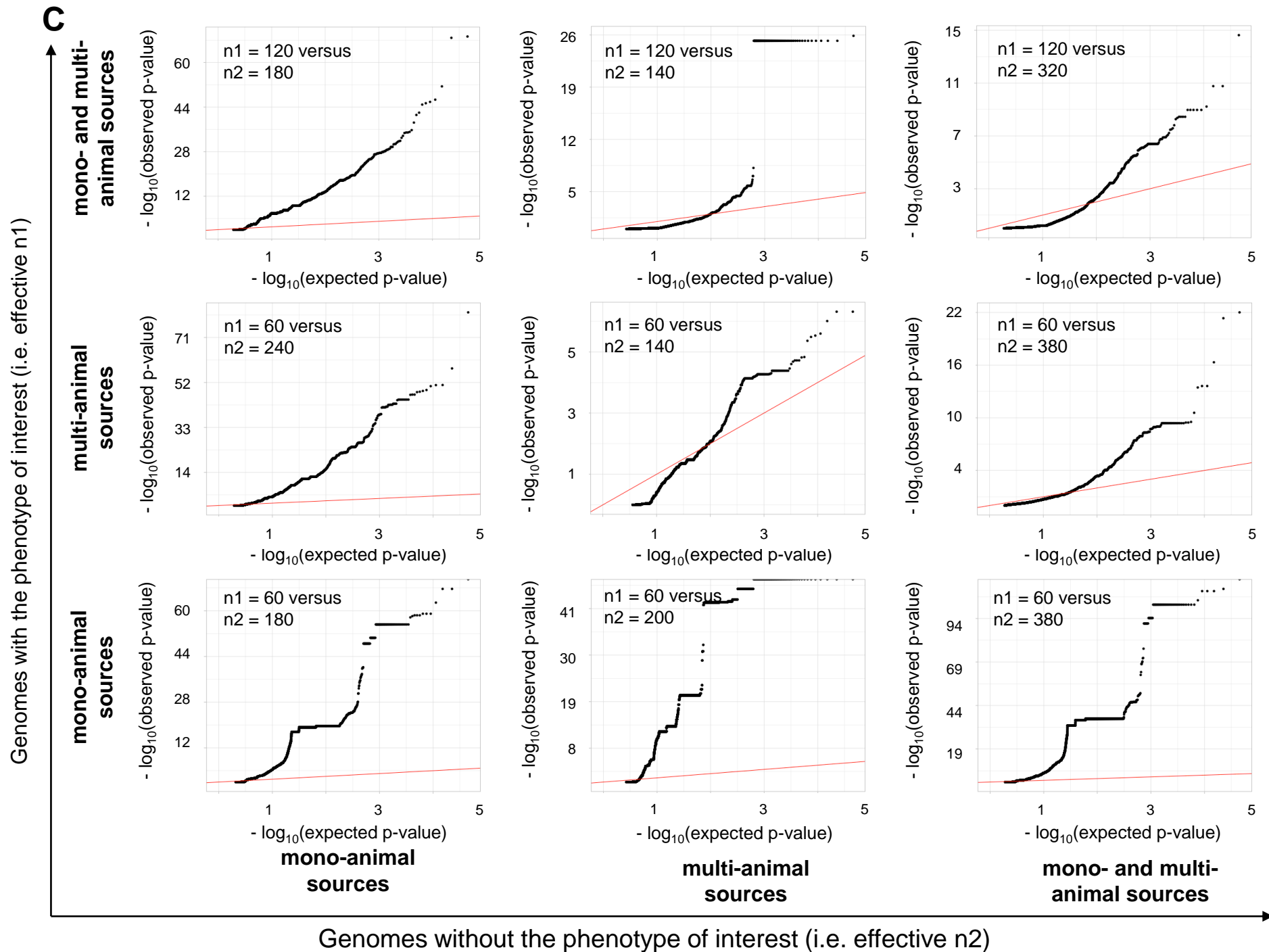

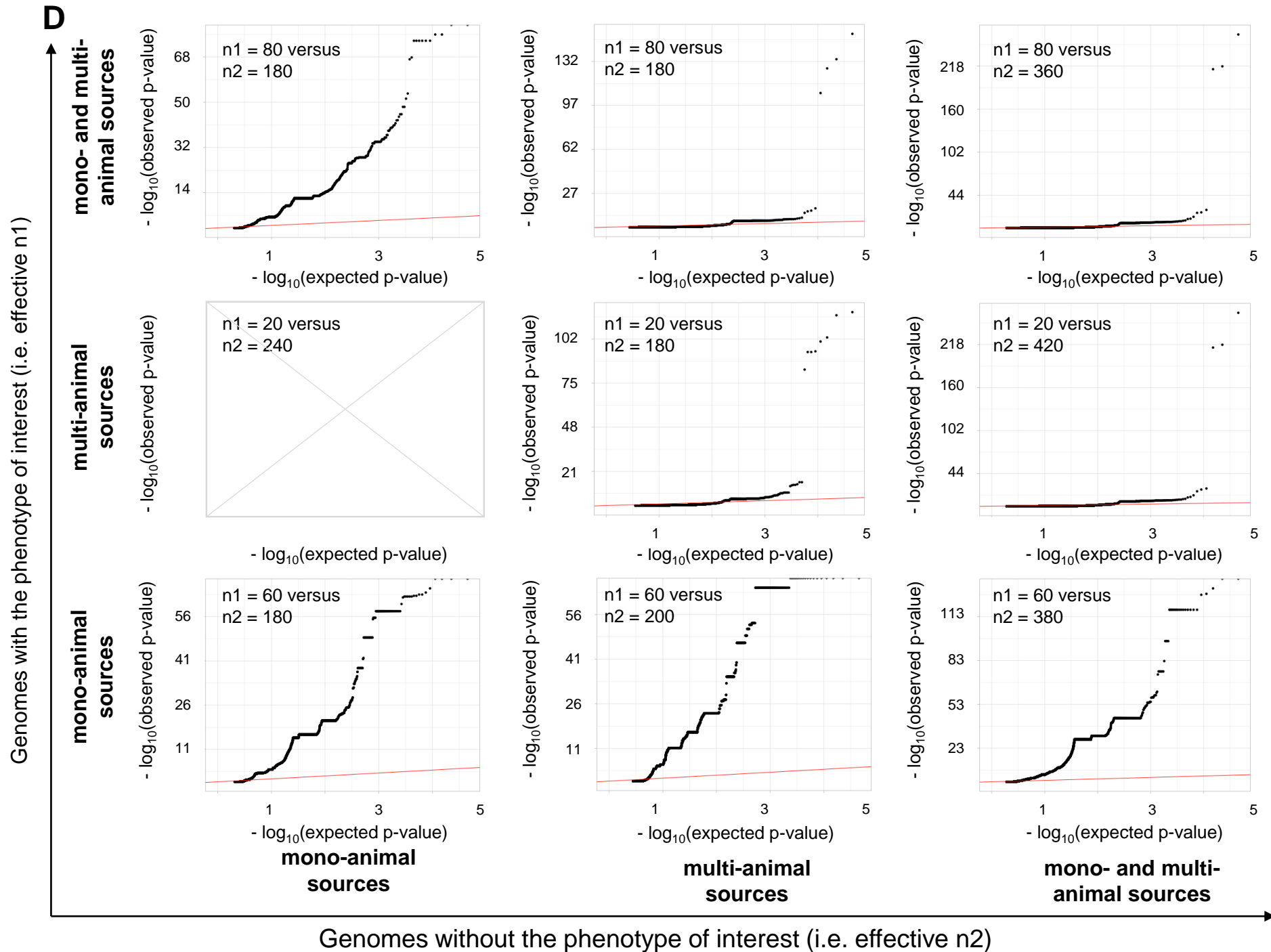

Supplement: Supplementary file 7 — Additional file 7. Quantile-Quantile (QQ) plots from microbial GWAS aiming to identify polygenicity during associations of accessory genes and coregenome variants excluding homologous recombination events of Salmonella enterica subsp. enterica serovars (n = 440) with avian (A), bovine (B), swine (C) and fish (D) sources. The samples were assigned to potential mono- and multi-animal sources based on a curated and synthetic version of Enterobase. The absence of GEMMA convergence is represented by a cross. The red line (i.e. - log10(observed p-values) = - log10(expected p-values)) corresponds to the reference line reflecting the level of population structure correction. [file 12864_2019_6188_MOESM7_ESM.pdf]
